# Characterisation of Four LIM Protein-Encoding Genes Involved in Infection-Related Development and Pathogenicity by the Rice Blast Fungus *Magnaporthe oryzae*

**DOI:** 10.1371/journal.pone.0088246

**Published:** 2014-02-05

**Authors:** Ya Li, Xiaofeng Yue, Yawei Que, Xia Yan, Zhonghua Ma, Nicholas J. Talbot, Zhengyi Wang

**Affiliations:** 1 State Key Laboratory for Rice Biology, Institute of Biotechnology, Zhejiang University, Hangzhou, Zhejiang, China; 2 School of Biosciences, University of Exeter, Exeter, Devon, United Kingdom; Rutgers University, United States of America

## Abstract

LIM domain proteins contain contiguous double-zinc finger domains and play important roles in cytoskeletal re-organisation and organ development in multi-cellular eukaryotes. Here, we report the characterization of four genes encoding LIM proteins in the rice blast fungus *Magnaporthe oryzae*. Targeted gene replacement of either the paxillin-encoding gene, *PAX1*, or *LRG1* resulted in a significant reduction in hyphal growth and loss of pathogenicity, while deletion of *RGA1* caused defects in conidiogenesis and appressorium development. A fourth LIM domain gene, *LDP1*, was not required for infection-associated development by *M. oryzae*. Live cell imaging revealed that Lrg1-GFP and Rga1-GFP both localize to septal pores, while Pax1-GFP is present in the cytoplasm. To explore the function of individual LIM domains, we carried out systematic deletion of each LIM domain, which revealed the importance of the Lrg1-LIM2 and Lrg1-RhoGAP domains for Lrg1 function and overlapping functions of the three LIM domains of Pax1. Interestingly, deletion of either *PAX1* or *LRG1* led to decreased sensitivity to cell wall-perturbing agents, such as Congo Red and SDS (sodium dodecyl sulfate). qRT-PCR analysis demonstrated the importance of both Lrg1 and Pax1 to regulation of genes associated with cell wall biogenesis. When considered together, our results indicate that LIM domain proteins are key regulators of infection-associated morphogenesis by the rice blast fungus.

## Introduction

The LIM domain is named after the three proteins (**L**in-11, **I**sl-1 and **M**ec-3) and consists of two tandemly-repeated zinc fingers within a conserved domain of 50-60 amino acids with consensus sequence CX_2_CX_16-23_HX_2_CX_2_CX_2_CX_16-21_CX_2_(C/H/D), where X denotes any amino acid; and/indicates alternatives [1]–[7]. However, unlike the DNA-binding function of many zinc fingers, the LIM domain does not bind DNA, but instead mediates specific protein-protein interactions, acting as a conserved scaffold to recognize diverse target proteins [5], [7]–[10]. LIM proteins regulate cell adhesion and motility, cytoskeleton organization, cell fate determination, and organ development (for review see Zheng and Zhao 2007). Considerable diversification of LIM protein function has occurred in multi-cellular eukaryotes [11] and the domain has been proposed to have been significant in the emergence of metazoa [12].

Many LIM proteins contain additional functional domains, such as homeodomains, RhoGAP domains and protein kinase activity [11], [13]. LIM proteins can therefore be classified into four groups according to the arrangement and position of LIM and other domains [7], [14]. Group 1 LIM proteins consists of LHX (LIM homeobox) proteins and nuclear LMO (LIM-domain-only) proteins, which localize to the nucleus and act as transcription factors or co-factors to mediate protein-protein interactions and thereby regulate gene expression. Group 2 constitutes LMO proteins consisting of two or more LIM domains, but unlike nuclear LMOs, proteins in this group are present in the cytoplasm or nucleus or can shuttle between compartments to regulate gene expression. Group 3 contains paxillin, zyxin, testin and enigma, which possess additional functional domains such as LD (leucine-aspartate repeat), ATD (actin-target domain) and PDZ (first letters of three proteins **P**SD95, **D**lg1 and **Z**o-1). In addition to LIM domains, proteins in Group 4 contain mono-oxygenase or kinase motifs that distinguish them from Group 3 [15], [16]. The conserved Group 3 paxillin proteins in animals consist of four characterized LIM domains at C-termini and an additional five LD motifs at the N-terminus [17], [18]. Paxillin serves as an adapter protein, mediating signal transduction from the extracellular matrix to focal adhesions and the actin cytoskeleton [11], [19]. Previous studies showed that C-terminal LIM domains in paxillin are involved in binding the protein tyrosine phosphatase PTP-PEST to target the protein to focal adhesions, and also to bind α- and γ-tubulin to direct an interplay between actin filaments and microtubules [20]–[22]. Through its LD motifs at N-termini, paxillin interacts with actopaxin (a member of the parvin family of focal-adhesion proteins), ILK (integrin-linked kinase), FAK (focal adhesion kinase), PKL (paxillin kinase linker) and vinculin to regulate Rho GTPase signaling and focal adhesion turnover [20], [21], [23], [24]. However, no LD motif has been discovered in the paxillin equivalent of yeasts and filamentous fungi, and only two or three LIM domains are present [25], [26]. In *Saccharomyces cerevisiae*, the paxillin homologue ScPxl1 coordinates Cdc42 and Rho1 function during polarized growth by directly binding to Rho1-GDP [27]. In *Schizosaccharomyces pombe*, SpPxl1 modulates Rho1 GTPase signaling and plays a role in formation and contraction of the actomyosin ring during cytokinesis by interaction with Rho1, Myo2 and Cdc15 [26], [28]. In *Ashbya gossypi*, the paxillin-like protein AgPxl1 plays a role in apical branching in hyphae [29]. Together with Rho-GTPases and the formin protein AgBni1, AgPxl1 also regulates spore length and spore wall integrity by directly interacting with AgRho1a and AgRho1b [30].

Two LIM proteins, Lrg1 and Rga1, which contain several LIM domains at the N-terminus and an extra RhoGAP domain at the C-terminus, have been identified in yeasts and the filamentous fungus *Neurospora crassa*. In *S. cerevisiae*, ScLrg1 is highly expressed in sporulating cells and may play a role during mating [31]. ScLrg1 has a specialized RhoGAP domain and negatively regulates 1, 3-β-glucan synthesis leading to an increase in 1, 3-β-glucan deposition in *Δlrg1* strains. It is therefore involved in the PKC1-mediated cell integrity pathway [32]–[34]. Disruption of *ScLRG1* in haploid cells results in enhanced invasive growth and a strain-specific ‘clustered’ phenotype that is a consequence of failed separation of mother and daughter cells in strain ∑1278b [35]. In addition, ScLrg1 locally inhibits cell wall synthesis to aid in the close apposition of the plasma membranes of mating cells [34]. *S. cerevisiae* ScRga1 controls the activity of Cdc42, which in turn controls the magnitude of signaling in the pheromone pathway via Ste20 [36]. In *N. crassa*, NcLrg1 acts as a Rho1-specific GAP affecting several output pathways of Rho1, which regulates polar tip growth and is involved in determining the size of the hyphal compartments by localizing to hyphal tips and sites of septation via its three LIM domains [37]. It has also been reported that accumulation of NcLrg1 is dependent on a functional actin cytoskeleton and active growth, and is influenced by the opposing microtubule-dependent motor proteins dynein and kinesin-1 in *N. crassa* [37].

To date, a large number of LIM proteins have been identified and characterized in plants, animals, but only a limited number of fungi. LIM proteins have not, for instance, been characterized in phytopathogenic fungi. *Magnaporthe oryzae*, the causal agent of rice blast disease, has emerged as a model for understanding molecular mechanisms of plant–pathogen interactions due to its molecular and genetic tractability [38]–[40]. *M. oryzae* uses specialized appressoria to penetrate the plant cuticle and then spreads within host cells as bulbous invasive hyphae, which ultimately erupt as aerial conidiophores to disseminate spores of the fungus to new host plants [38]. Previously, we reported that a LIM domain-binding protein, Ldb1, is necessary for vegetative growth, infection-related morphogenesis and pathogenicity of the rice blast fungus, however, we could not detect a direct interaction between Ldb1 and putative LIM proteins (Pax1, Lrg1, Rga1/Lrg2 and Ldp1) in yeast two-hybrid assays [41]. We speculated that a large protein complex is associated with the action of Ldb1. We therefore set about characterizing the four putative LIM proteins in *M. oryzae* to determine the role of this signaling pathway in the fungus and to shed light on the wider role of the LIM domain in fungal development and pathogenicity. Our results reveal important roles for the LIM domain family of protein in rice blast disease.

## Results

### Identification of four genes putatively encoding LIM proteins in *M. oryzae*

**Figure 1:**
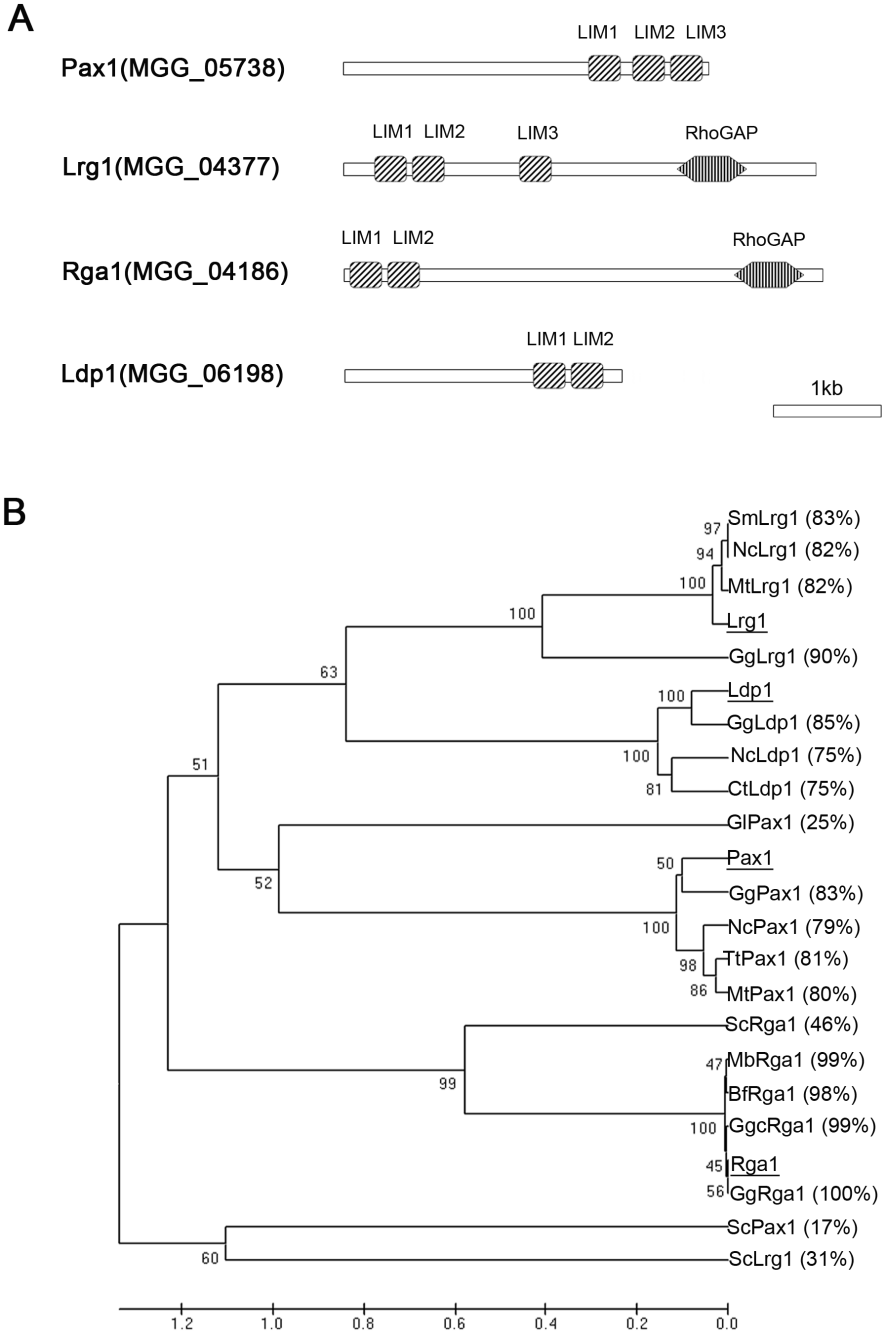
Four predicted LIM proteins in *M. oryzae*. A. Domain structures and position of the four predicted LIM proteins in *M. oryzae*; B. Phylogenetic analysis among the LIM domain regions from different species. The identity of each *M. oryzae* LIM domain region to its homologs was analyzed by DNAMAN software and indicated in brackets. The phylogenetic tree was constructed using the software MEGA4. Protein abbreviations corresponding to species names and predicted proteins (GenBank accession numbers) are: Pax1, *M. oryzae* paxillin (XP_003710649); GgPax1, *Gaeumannomyces graminis* paxillin (EJT81269); TpPax1, *Thielavia terrestris* paxillin (XP_003652151); MtPax1, *Myceliophthora thermophila* paxillin (XP_003659433); NcPax1, *Neurospora crassa* paxillin (XP_964072); ScPax1, *Saccharomyces cerevisiae* Pxl1 (CAY81167); GglPax1, *Gallus gallus* paxillin (NP_990315); Lrg1, *Magnaporthe oryzae* Lrg1 (XP_003713492); GgLrg1, *Gaeumannomyces graminis* Lrg1 (EJT73429); SmLrg1, *Sordaria macrospora* Lrg1 (XP_003351045); NcLrg1, *N. crassa* Lrg1 (CAE76522); MtLrg1, *Myceliophthora thermophila* Lrg1 (XP_003659561); ScLrg1, *Saccharomyces cerevisiae* Lrg1 (NP_010041); Rga1, *Magnaporthe oryzae* Rga1 (XP_003719637); GgRga1, *G. graminis* Rga1 (EJT77859); MbRga1, *Marssonina brunnea* Rga1 (EKD19800); GgcRga1, *Glomerella graminicola* Rga1 (EFQ33209); BfRga1, *Botryotinia fuckeliana* Rga1 (XP_001551485); ScRga1, *S. cerevisiae* Rga1 (CAY86414); Ldp1, *Magnaporthe oryzae* Ldp1 (XP_003712085); GgLdp1, *Gaeumannomyces graminis* Ldp1 (EJT77591); NcLdp1, *N. crassa* Ldp1 (XP_960915); CtLdp1, *Chaetomium thermophilum* Ldp1 (EGS18643).

Bioinformatics analysis revealed four genes putatively encoding LIM domain-containing proteins in the *M. oryzae* genome as shown in Fig. 1A. These were named Pax1 (*M*. *oryzae* paxillin homolog), Ldp1 (*M*. *oryzae* LIM domain-containing protein), Lrg1 (*M. oryzae* LIM and RhoGAP) and Rga1 (*M*. *oryzae* Rho GTPase activator) (termed Lrg2 in Li et al. 2010a). Based on the phylogenetic analysis of LIM domain regions of LIM proteins from different fungal species, the four predicted LIM proteins in *M. oryzae* could be divided into four distinct clades as shown in Fig. 1B. The proteins were diverse in amino acid identity, but the LIM domain regions were more highly conserved. For instance, the Pax1 LIM domains were 83, 81, 80 and 79% identical to those in predicted paxillins from *Gaeumannomyces graminis*, *Thielavia terrestris*, *Myceliophthora thermophila* and *N*. *crassa*, but only 17% identical to paxillin from *S*. *cerevisiae* (Fig. 1B).

### Targeted deletion of *PAX1*, *LRG1*, *RGA1* and *LDP1* in *M. oryzae*

To understand the function of genes encoding LIM proteins in *M. oryzae*, targeted gene deletion mutants of each LIM domain protein-encoding gene were generated and confirmed by Southern blot analysis (Fig. S1). Gene deletion mutants of *RGA1* and *LDP1* were generated from the wild-type strain Guy11 [42]. However, we were unable to obtain *LRG1* and *PAX1* gene deletion mutants from Guy11 after examining more than three hundred transformants from various independent transformation experiments. We therefore used the isogenic *Δku70* and *Δku80* mutants of Guy11 as recipient strains for deletion of *LRG1* and *PAX1* because they show high rates of homologous recombination [43], [44]. Hereafter, both *Δku70* (Ku70) and *Δku80* (Ku80) are described as “wild type” strains due to their phenotypic similarity to Guy11 [43], [44]. As listed in Table S1, we obtained three independently generated targeted gene replacement mutants for each of the four LIM protein genes, and selected LP55 (*Δpax1*), LR80 (*Δlrg1*), LG25 (*Δrga1*) and LD17 (*Δldp1*) for detailed phenotypic analysis.

### Deletion of *PAX1* or *LRG1* significantly impaired vegetative growth of *M. oryzae*

**Figure 2:**
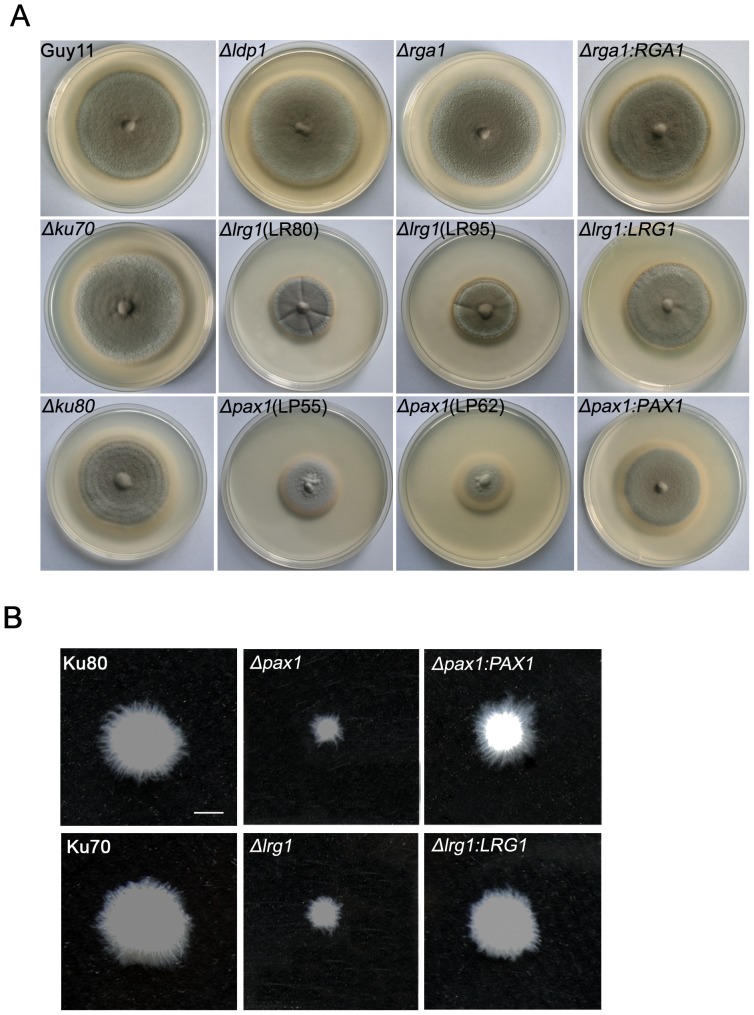
Vegetative growth of the *Δpax1* and *Δlrg1* mutants was significantly impaired in *M. oryzae*. **A**. The vegetative growth of the *Δpax1* and *Δlrg1* mutants was significantly impaired. Colonies of the *Δldp1* (LD17), *Δrga1* (LG25), *Δlrg1* (LR80 and LR95) and *Δpax1* (LP55 and LP62) mutants were formed on CM plates at 25°C for 10 d; **B**. The *Δpax1* (LP55) and *Δlrg1* (LR80) mutants grew slowly in liquid CM medium and formed small mycelium masses compared with those of wild-type strains. Wild-type strains: Guy11, Ku70 (*Δku70*) and Ku80 (*Δku80*). Gene complementation strains: GC22 (*Δrga:RGA1*), RC38 (*Δlrg1:LRG1*) and PC20 (*Δpax1:PAX1*). Bar  =  5 mm.

To evaluate the role of *M. oryzae* LIM proteins in hyphal growth, strains were grown on solid complete medium (CM) for 10 days and colony diameters measured (Fig. 2A; Table 1). The *Δpax1* and *Δlrg1* mutants displayed a significant reduction in growth with diameter of (3.0±0.1) and (3.5±0.1) cm, respectively, compared with the wild type strains Ku80 of (6.9±0.1) cm and Ku70 of (6.9±0.1) cm (t-test, P<0.01) (Fig. 2A; Table 1). Similarly, when incubated in liquid CM medium for 48 h, the mutants grew slowly compared to the isogenic wild-type (Fig. 2B). Growth defects were complemented by re-introduction of the *PAX1* and *LRG1* genes into *Δpax1* and *Δlrg1* mutants, respectively (Fig. 2; Table 1). No obvious growth difference was observed in *Δrga1* or *Δldp1* mutants as shown in Fig. 2 and Table 1. These results suggest that both *PAX1* and *LRG1* are involved in hyphal growth in *M. oryzae*.

**Table 1:** Phenotypic analysis of LIM protein mutants in *M. oryzae*.

Strain	Growth (cm)^a^	Conidiation (×10^4/plate^)^b^	Appresorium (%)^c^		Penetration(%)^d^
			GB	OE	
Guy11	6.8±0.1a^e^	99.7±6.0a	97.0±3.6a	96.7±3.5a	86.7±3.2a
*Δrga1*	6.8±0.1a	95.7±10.7a	13.3±3.8b	95.7±2.5a	83.3±4.5a
*Δrga:RGA1*	6.8±0.1a	98.7±8.3a	96.7±4.2a	95.0±3.0a	86.0±4.0a
*Δldp1*	6.9±0.1a	93.3±7.6a	95.7±2.6a	94.5±4.5a	85.0±3.0a
Ku80	6.9±0.1a	93.3±1.5a	95.7±3.5a	93.7±2.1a	82.7±3.1a
*Δpax1*	3.0±0.1d	0	0	0	0
*Δpax1:PAX1*	5.9±0.1b	62.3±3.1b	96.3±1.5a	91.7±4.a	82.7±5.0a
Ku70	6.8±0.1a	95.7±1.5a	94.7±4.0a	92.3±4.5a	82.3±3.1a
*Δlrg1*	3.5±0.1c	3.3±1.5c	0	0	0
*Δlrg1:LRG1*	5.9±0.1b	65.3±4.5b	94.7±5.1a	93.7±3.5a	84.7±1.5a

**a**. The diameter of colonies grown on CM plates at 25°C for 10 d. **b**. The conidia washed from the 15d-old CM cultures. **c**. Percentage of appressorium formation on GB (GelBond) surfaces incubated for 24 h or OE (onion epidermis) surfaces incubated for 48 h at 25°C. **d**. Percentage of invasive hyphae formation from appressoria incubated on OE for 24 h at 25°C. More than 300 spores or appressoria were counted for each strain. **e**. Different letters after mean values indicated significant difference at P-value of 0.05. Data were calculated from three independent experiments conducted in triplicates. Strains: Guy11, LG25 (*Δrga1*), GC22 (*Δrga:RGA1*), LD17 (*Δldp1*), Ku80, LP55 (*Δpax1*), PC20 (*Δpax1:PAX1*), Ku70, LR80 (*Δlrg1*) and RC38 (*Δlrg1:LRG1*).

### *PAX1* and *LRG1* are involved in conidiogenesis and septation in *M. oryzae*

**Figure 3:**
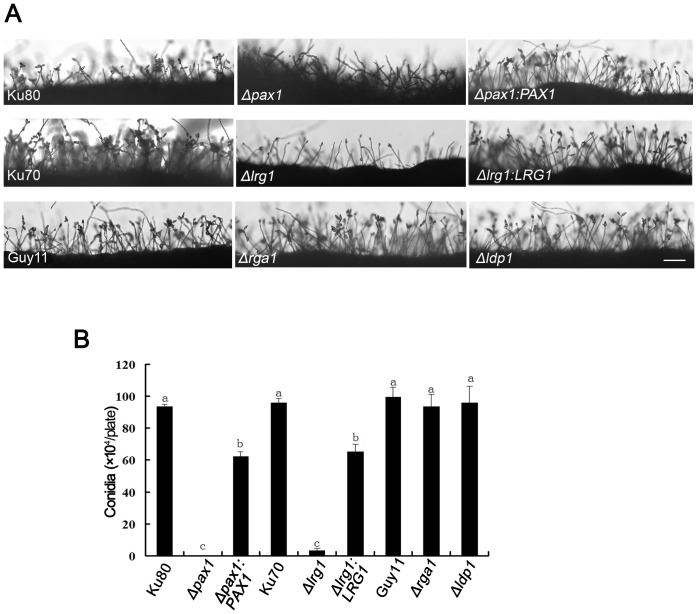
The *Δpax1* mutants were unable to produce conidia and deletion of *LRG1* resulted in significant reduction in conidiation in *M. oryzae*. **A**. Microscopic observation of conidia and conidiophores of the cultures on solid CM medium. Bar  =  0.0031 mm; **B**. Bar chart showed conidial production of different strains on solid CM medium. Conidia per plate were carefully harvested from 15d-old cultures. Data were calculated from three independent experiments conducted in triplicates. Different small letters indicated significant difference at P-value of 0.05. The strains used for the analysis were Guy11, Ku80, Ku70, LP55 (*Δpax1*), LR80 (*Δlrg1*), LD17 (*Δldp1*), LG25 (*Δrga1*), PC20 (*Δpax1:PAX1*) and RC38 (*Δlrg1:LRG1*).

**Figure 4:**
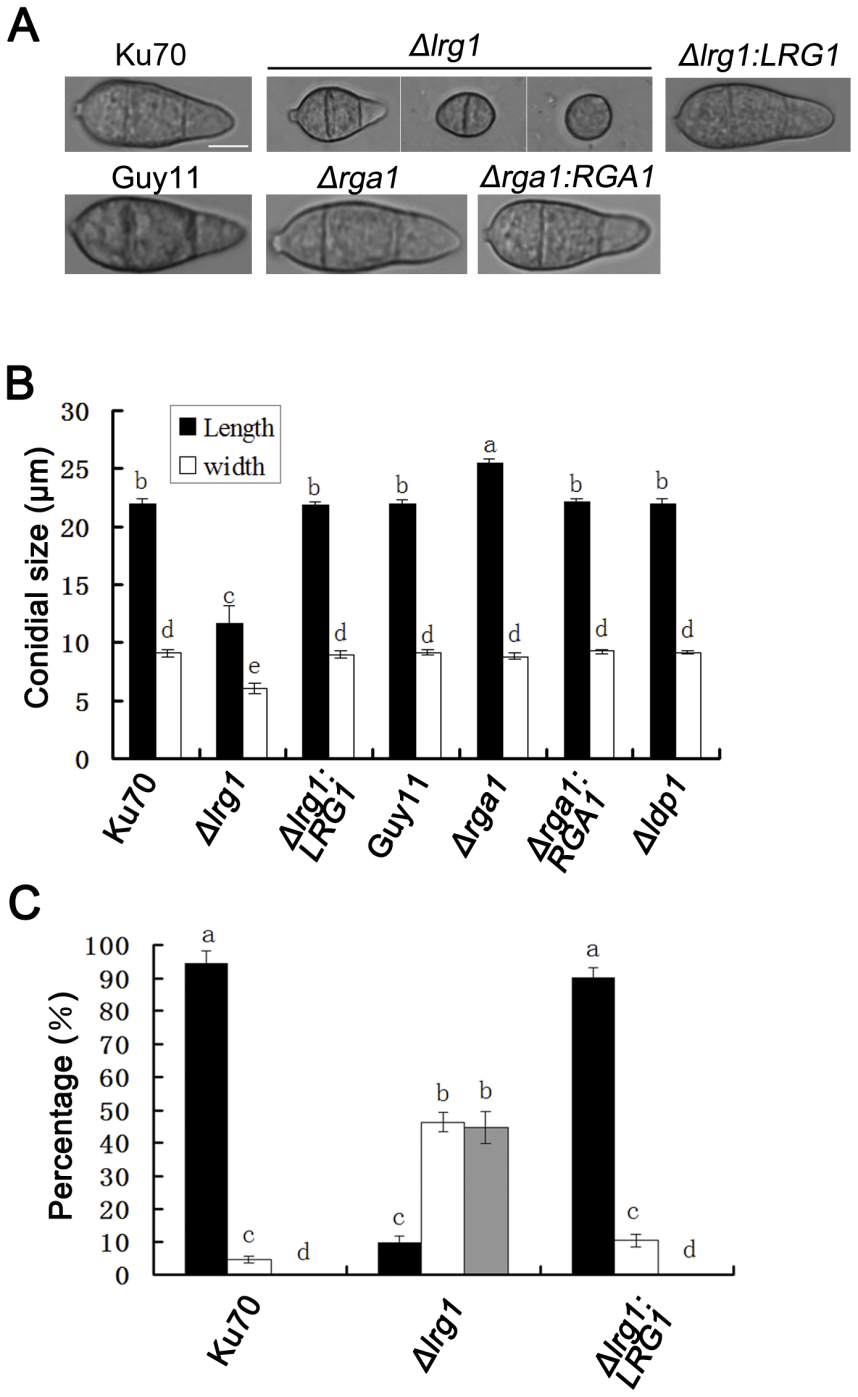
The *Δlrg1* and *Δrga1* mutants produced morphologically abnormal conidia. **A**. Microscopic observation of conidia from different strains: Guy11, Ku70, LR80 (*Δlrg1*), RC38 (*Δlrg1:LRG1*), LG25 (*Δrga1*) and GC22 (*Δrga1:RGA1*). Bar  =  5 µm; **B**. Bar chart showed conidial size in length and width of different strains. LG25 (*Δrga1*) produced significantly longer conidia than the wild-type strain Guy11, whereas LR80 (*Δlrg1*) generated smaller conidia both in length and width compared to Ku70; **C**. Proportions of conidia of the *Δlrg1* mutant (LR80) with 0, 1 or 2 septa. All data in **B** and **C** were calculated from three independent experiments conducted in triplicates. Different letters indicated significant difference at P-value of 0.05.

To analyze the roles of each LIM protein in asexual sporulation, we quantitatively measured conidial production by harvesting conidia from 15-day-old cultures of *M. oryzae*. The *Δpax1* mutant was unable to produce any conidia (Table 1; Fig. 3A and B), while the *Δlrg1* mutant produced significantly reduced numbers of conidia with (3.3±1.5) ×10^4^ spores/plate, which was only 3% of the number of spores generated by the wild-type strain Ku70 (95.7±1.5) ×10^4^ spores/plate (Table 1; Fig. 3B). Re-introduction of *PAX1* and *LRG1* into *Δpax1* and *Δlrg1* mutants complemented this phenotype, respectively (see Table 1; Fig. 3). Microscopy revealed a reduction in aerial conidiophores in *Δpax1* and *Δlrg1* mutants (Fig. 3A). Conidia produced by the *Δlrg1* mutant were significantly smaller with average length and width of (11.66±1.47) µm and (6.01±0.43) µm, respectively, compared to the wild type Ku70 with a length of (21.97±0.35) µm and a width of 9.10±0.27 µm (t-test, P<0.01) (Fig. 4A and B). By contrast, the *Δrga1* mutant produced elongated conidia with (25.51±0.34) µm in length compared to the wild type Guy11 with a length of (21.98±0.35) µm (Fig. 4A and B). The *Δlrg1* mutant also produced conidia with 0, 1 or 2 septa (Fig. 4A). The proportion of 0, 1 and 2 septal conidia of the *Δlrg1* mutant was 44.6%, 45.3% and 9.8%, respectively. These effects were all complemented by re-introduction of wild type alleles of each gene as shown in Fig. 4. No effect on sexual reproduction was observed by loss of LIM domain genes (Fig. S2), suggesting that these proteins are not required for sexual sporulation in *M. oryzae*.

### *Δlrg1* and *Δpax1* mutants are unable to form appressoria

**Figure 5:**
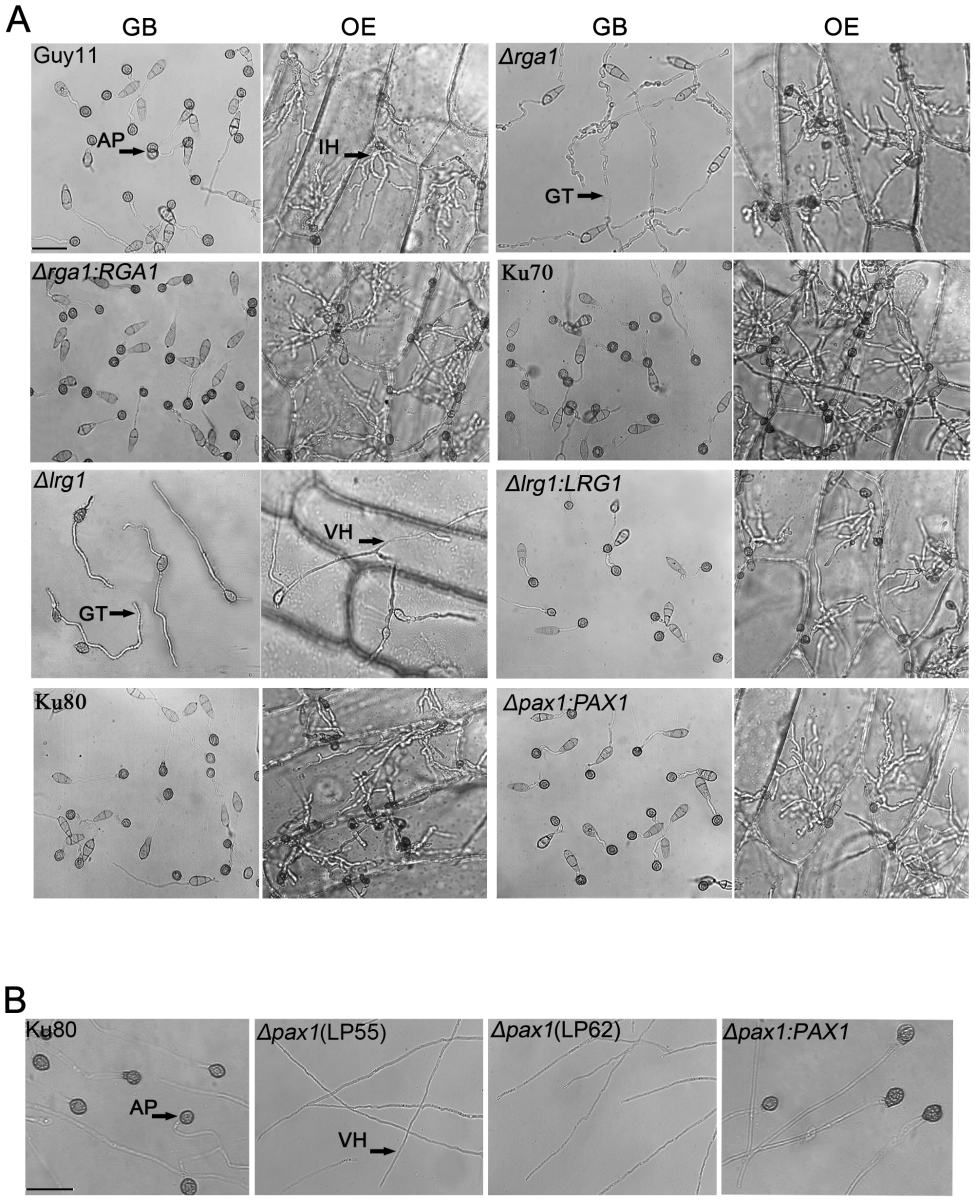
Appressorium formation and penetration assays of the *Δpax1* and *Δlrg1* mutants. **A**. Appressorium formation on GelBond (GB) hydrophobic surfaces at 25°C for 24 h and appressorium mediated-penetration on onion epidermis (OE) at 25°C for 48 h. The strains were Guy11, Ku80, Ku70, PC20 (*Δpax1:PAX1*), LR80 (*Δlrg1*), RC38 (*Δlrg1:LRG1*), LG25 (*Δrga1*) and GC22 (*Δrga1:RGA1*). AP, appressorium; IH, invasive hypha; GM, germ tube; VH, vegetative hypha; **B**. Appressorium formation induced from mycelium of Ku80, LP55 (*Δpax1*), LP62 (*Δpax1*), PC20 (*Δpax1:PAX1*) on GB surface at 25°C for 24 h. Bars in **A** and **B**  =  25 µm.

To understand the roles of the LIM proteins in appressorium-mediated plant infection by the blast fungus, conidial suspensions of *Δlrg1*, *Δrga1* and *Δldp1* mutants were incubated on hydrophobic GelBond to induce appressorium formation, and on onion epidermis to observe tissue penetration. The wild-type strains Guy11, Ku70, Ku80 and the complementation strains *Δrga1:RGA1*, *Δlrg1:LRG1*, *Δpax1:PAX1* produced normal melanized appressoria on both plastic and onion epidermis surfaces and formed invasive hyphae in onion epidermal cells (Table 1; Fig. 5A). By contrast, the *Δlrg1* mutant failed to form appressoria or invasive hyphae (Table 1; Fig. 5A). In *Δrga1* mutants only 13.3% of conidia formed appressoria on plastic hydrophobic surface, but *Δrga1* mutants produced appressoria and penetrated plant cells normally (Table 1; Fig. 5A), indicating that *RGA1* may be involved in surface recognition and sensing in *M. oryzae*, but in a manner that does not affect morphogenesis on the plant surface. *Δldp1* mutants formed appressoria and penetrated normally. Given that *Δpax1* mutants are unable to produce conidia, we prepared hyphal suspensions to induce appressorium formation on plastic hydrophobic surface. We did not observe any appressorium formation, whereas Ku80 and the complemented strain (*Δpax1:PAX1*) produced normal appressoria when prepared in the same way (Fig. 5B). We conclude that *LRG1* and *PAX1* are essential for appressorium formation and penetration in *M. oryzae*.

### *Δpax1* and *Δlrg1* mutants are unable to cause rice blast disease

**Figure 6:**
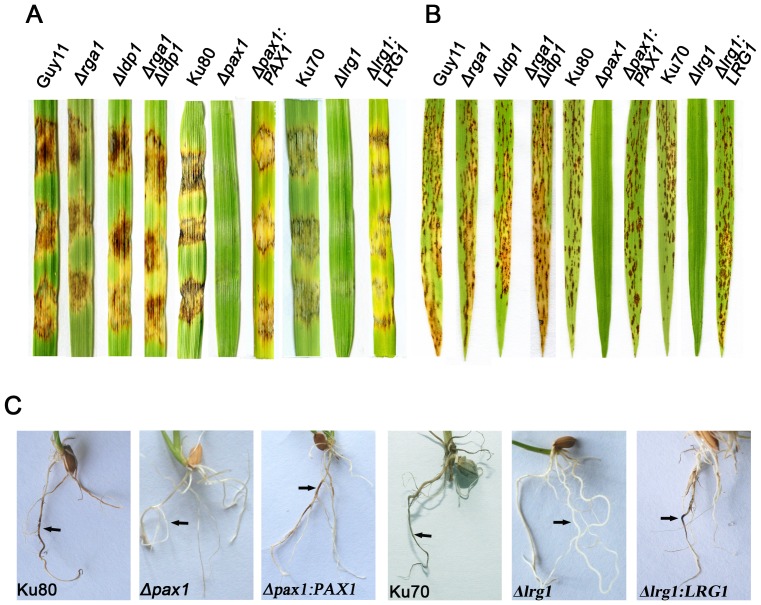
Both *Δlrg1* and *Δrga1* mutants are nonpathogenic on susceptible hosts. **A**. Barley leaf segments were inoculated with the mycelia harvested from liquid cultures of different strains. **B**. Rice seedling infection assays by spray-inoculating with conidial suspension (1×10^5^ conidia ml^−1^) of all strains except LP55 (*Δpax1*). Mycelium segment suspension of the *Δpax1* mutant was used for inoculation due to its inability to produce conidia. **C**. Rice roots infected with mycelium plugs from different strains. Arrows indicate necrotic lesions. Photographs were taken after inoculation for 7 d. The strains were Guy11, Ku80, Ku70, LP55 (*Δpax1*), PC20 (*Δpax1:PAX1*), LR80 (*Δlrg1*), RC38 (*Δlrg1:LRG1*), LG25 (*Δrga1*) and LD17 (*Δldp1*), AD27 (*Δrga1Δldp1*).

To determine whether LIM proteins are involved in pathogenicity of *M. oryzae*, we performed plant infection assays. In a cut-barley-leaf assay inoculated with mycelial fragments, *Δpax1* and *Δlrg1* mutants failed to produce blast disease symptoms, whereas *Δrga1* and *Δldp1* mutants and all complemented strains caused obvious disease (Fig. 6A). Similarly, when 2-week-old rice seedlings were spray-inoculated with conidial suspensions of different strains (or inoculated by mycelial suspension for *Δpax1* due to its lack of conidiation), *Δpax1* and *Δlrg1* mutants were non-pathogenic (Fig. 6B). We also performed rice root infection assays [45] and found that *Δpax1* and *Δlrg1* mutants were unable to cause disease (Fig. 6C).

### Lrg1 and Rga1 localize to septal pores in *M. oryzae*

**Figure 7:**
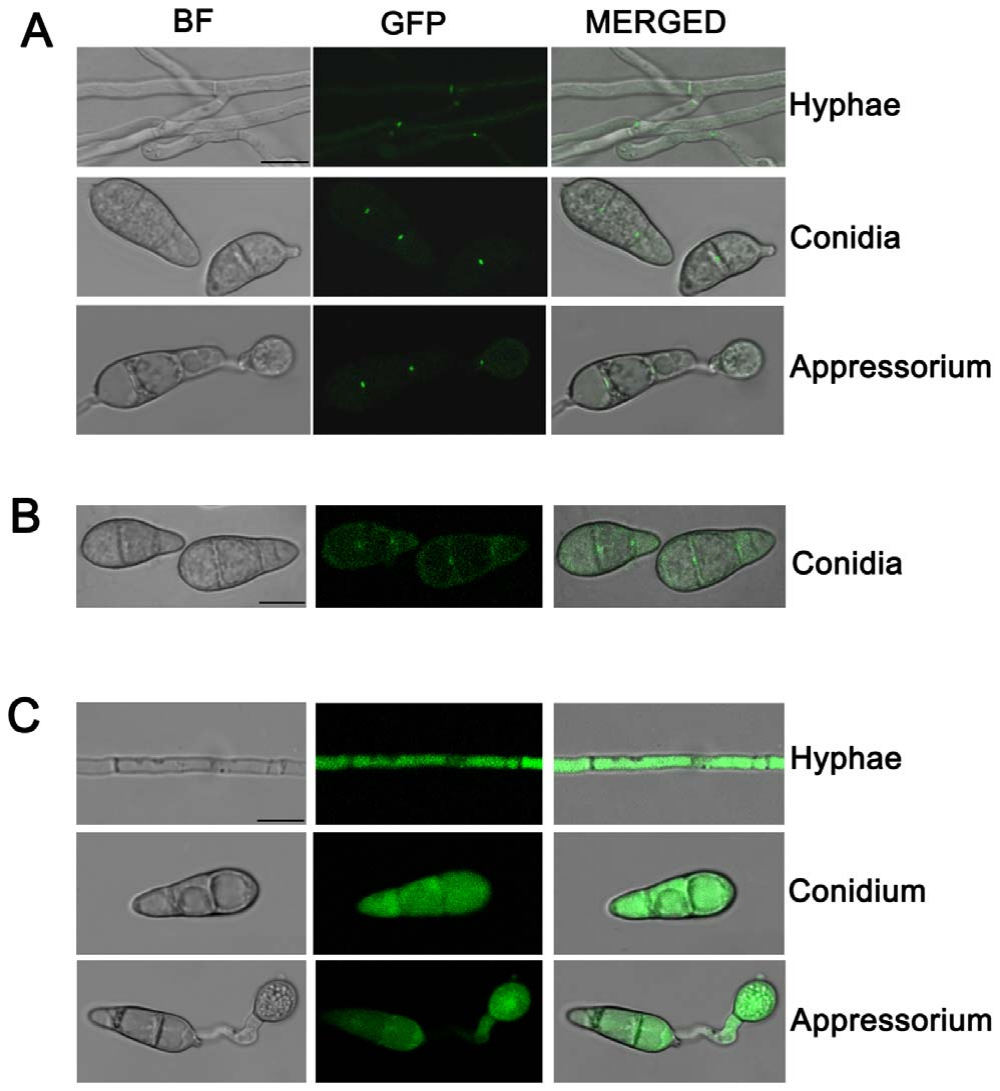
Subcellular localization of LIM proteins in *M. oryzae*. **A**. Septal pore localization of Lrg1-GFP in hyphae, conidia, and appressoria of the *Δlrg1:LRG1* strain (RC38); **B**. Septal pore localization of Rga1-GFP in conidia of the *Δrga1:RGA1* strain (GC22); **C**. Cytoplasmic localization of Pax1-GFP in hyphae, conidia, and appressoria of *Δpax1:PAX1* (PC20). Bars  =  10 µm.

To analyze the subcellular localization of *M. oryzae* LIM proteins, corresponding C-terminal GFP fusions were expressed in *Δpax1, Δlrg1* and *Δrga1* mutants. As shown in Table 1 and Figures 2–6, the resulting transformants complemented all mutant phenotypes confirming that they are functionally active proteins. In live cell imaging experiments we observed septal pore localization of both Lrg1- and Rga1-GFP fusions (Fig. 7A and B), whereas Pax1-GFP was observed throughout the cytoplasm (Fig. 7C). To visualize *LRG1* and *PAX1* expression during appressorium development, conidia of the strains RC38 (*Δlrg1:LRG1*) and PC20 (*Δpax1:PAX1*) were allowed to germinate on hydrophobic GelBond. Lrg1-GFP and Pax1-GFP expression was observed during initial stages of germination and appressorium formation but decreased during appressorium maturation (Fig. S3 and Fig. S4).

### Functional characterization of individual LIM domains of Lrg1 and Pax1

**Figure 8:**
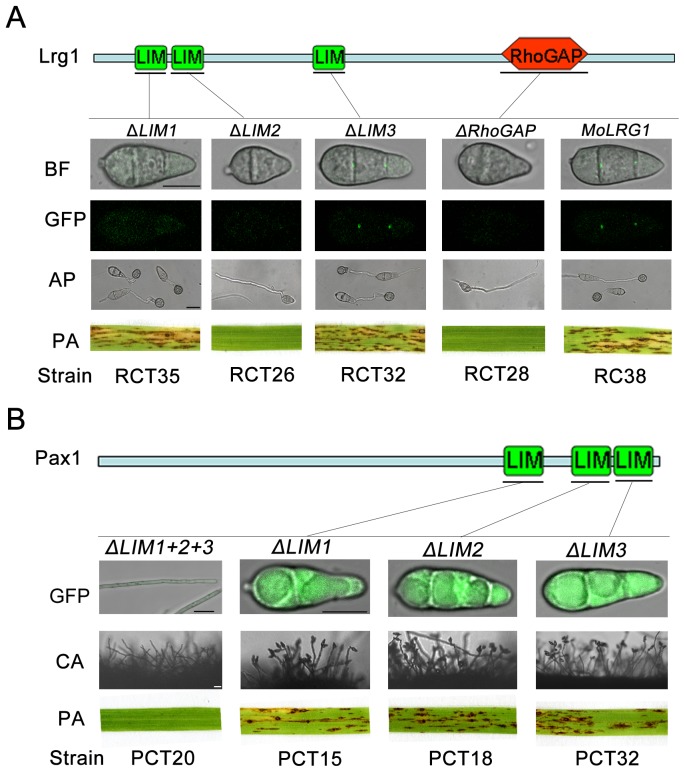
Functional analysis of the domains in Lrg1 and Pax1. **A**. Functional analysis of each domain in Lrg1. Either Lrg1-LIM2 or -RhoGAP was essential for appressorium formation, pathogenicity and proper protein localization. Lrg1-LIM1 was only required for Lrg1 localization to septum pores, whereas Lrg1-LIM3 was dispensable for Lrg1 function and localization. CON, conidium; AP, appressorium; PA, pathogenicity assay; **B**. Functional analysis of each domain in Pax1. Single deletion of the LIM domains in Pax1 didn't lead to any defects in conidiation and pathogenicity, while deletion of the region contained all the three LIM domains resulted in an inability to produce conidia and cause blast disease. CA, Conidiation. Bars  = 10 µm.

Lrg1 and Pax1 contain multiple LIM domains and Lrg1 contains an additional Rho-GAP domain (Fig. 1A). To determine the function of these individual domains we constructed alleles in which each domain was individually deleted and introduced these into the *Δlrg1* and *Δpax1* mutants. Deletion of either Lrg1-LIM2 or the Lrg1-RhoGAP domain resulted in an inability to overcome the defects of the *Δlrg1* mutant in conidiation, appressorium formation and pathogenicity. Furthermore, the septal pore localization of Lrg1-GFP was not observed (Fig. 8A), suggesting that both domains are necessary for correct localization and function of Lrg1. Deletion of the Lrg1-LIM1 domain affected localization but not the function of Lrg1, while deletion of the third LIM domain, Lrg1-LIM3 had no observable effect (Fig. 8A). Taken together, these results suggest that the LIM2 domain is the most critical for function of Lrg1.

We next investigated the function of each LIM domain of Pax1 (Fig. 8B). Deletion of any LIM domain singly had no discernable effect, whereas deletion of all three LIM domains impaired function of Pax1 completely (Fig. 8B). This suggests that the three LIM domains of Pax1 may have redundant or overlapping functions in infection-related morphogenesis.

### Deletion of *PAX1* or *LRG1* led to up-regulation of genes involved in cell wall biosynthesis and re-modeling

**Figure 9:**
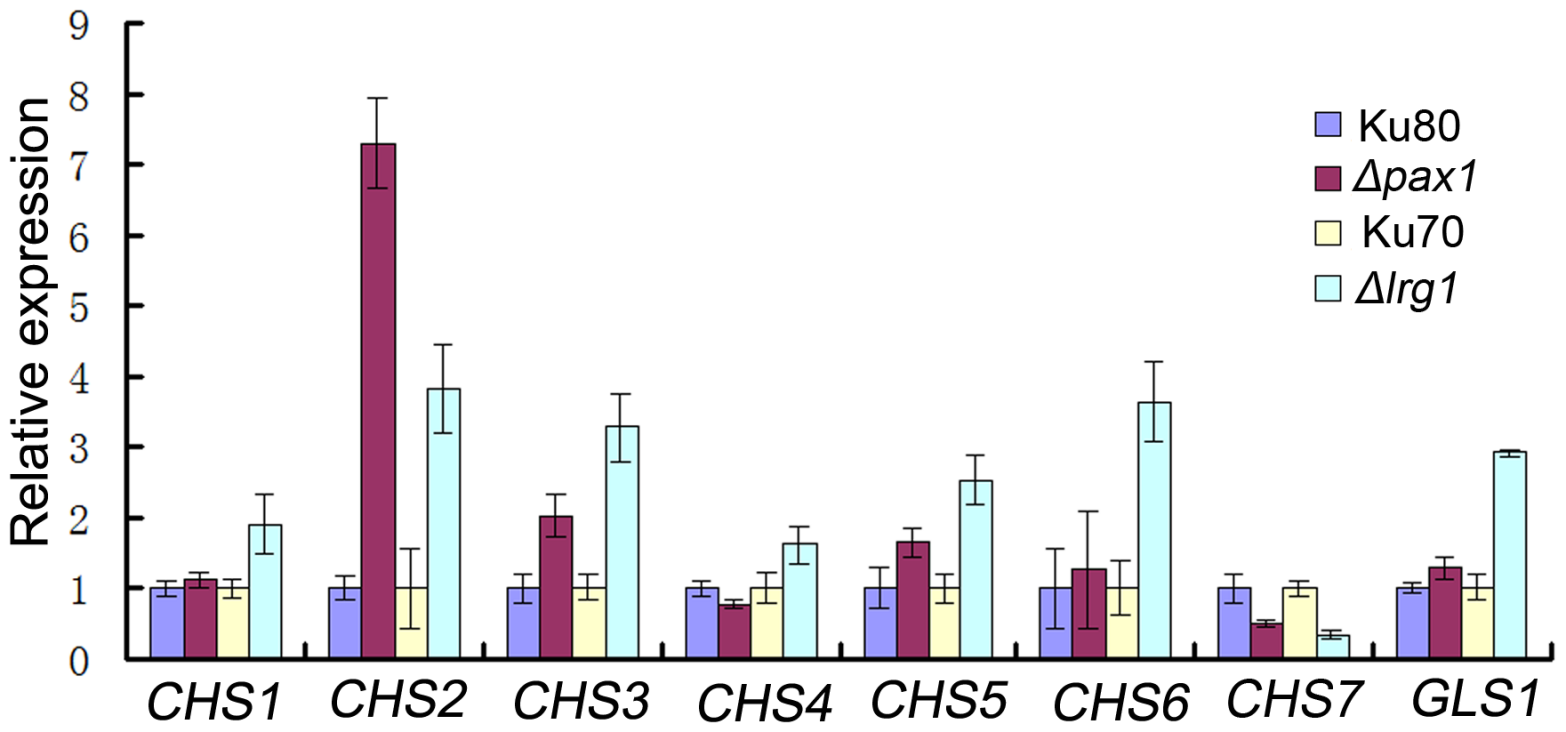
Expression analysis of several cell wall synthesis-related genes in *Δlrg1* and *Δpax1* mutants by qRT-PCR. All of the tested cell wall synthesis-related genes but not *CHS7* were up-regulated in either *Δlrg1* or *Δpax1* mutant. *CHS1*, MGG_01082; *CHS2*, MGG_04145; *CHS3*, MGG_09551, *CHS4*, MGG_09962; *CHS5*, MGG_13014; *CHS6*, MGG_13013, *CHS7*, MGG_06064; *GLS1*, MGG_00865. Ku70, *Δku70*; Ku80, *Δku80*; LR80, *Δlrg1*; LP55, *Δpax1*. Statistical data were calculated from three independent experiments conducted in triplicates.

To determine whether LIM proteins are associated with regulation of cell wall integrity in *M. oryzae*, given their roles in sporulation and appressorium development, we exposed each mutant to exogenous hyperosmotic concentrations of NaCl and sorbitol, or to agents associated with cell wall stress, Congo Red (CR), sodium dodecyl sulfate (SDS) and H_2_O_2_ (Table S3). *Δpax1* and *Δlrg1* mutants were more tolerant to 1 M NaCl, but showed no significant difference to 1.2 M sorbitol compared with the wild type strains Ku80 and Ku70, respectively (Table S3). On CM plates with 100 µg/ml Congo Red, a low inhibition rate of (1.2±0.5)% and (10.7±4.4)% was observed in *Δpax1* and *Δlrg1* mutants, respectively, comparing with (27.8±1.9)% in Ku80 and (26.9±5.3)% in Ku70, respectively (Table S3). Similarly, in CM plates with 0.05% SDS, inhibition rates of (45.0±2.5)% and (52.33±1.91)% for *Δpax1* and *Δlrg1* mutants were observed, respectively, which was significantly lower than (61.51±2.26)% of Ku80 and (64.3±4.6)% of Ku70 (Table S3), suggesting that deletion of either *PAX1* or *LRG1* led to a decreased sensitivity to these cell wall-perturbing agents. *Δrga1* and *Δldp1* mutants had similar sensitivity to Guy11 and all mutants showed normal sensitivity to H_2_O_2_ (data not shown). Pax1 and Lrg1 are therefore likely to play roles in the cellular response to osmotic and cell wall integrity. To test this idea, we conducted qRT-PCR to determine the expression levels of genes associated with cell wall synthesis including chitin and glucan synthases. This revealed that the majority of cell wall biosynthesis-related genes (*CHS1* to *CHS6* and *GLS1*) were up-regulated in the absence of *PAX1* and *LRG1* (Fig. 9). We conclude that both LIM domain proteins are involved in regulation of cell wall integrity in *M. oryzae.*

## Discussion

LIM proteins play key roles in cytoskeleton organization, organ development and cell fate determination in eukaryotes [7]. To date, a large number of LIM proteins have been characterized in animals, but very few have been reported in fungi, with reports in *S. cerevisiae* [25], [27], [31], [33], [34], [36], *Schizosaccharomyces pombe* [26], [28], *A. gossypii* [29], [30], and *N. crassa* [37]. In this study, we characterized four genes, *PAX1*, *LRG1*, *RGA1* and *LDP1* in the rice blast fungus *M. oryzae*. Targeted gene replacement analysis revealed that *Δpax1* mutants showed significantly reduced in vegetative growth and were unable to produce conidia and appressoria (Fig. 2, 3 and 5B). *Δpax1* mutants were also incapable of causing blast disease on susceptible hosts (Fig. 6). *Δlrg1* mutants were also impaired in vegetative growth and unable to form appressoria or cause disease (Fig. 2, 5 and 6). These results therefore suggest that both Pax1 and Lrg1 are involved in regulating vegetative growth, conidiation, appressorium formation and pathogenicity in *M. oryzae*. Deletion of *RGA1* resulted in only minor changes to conidial morphology and appressorium formation (Fig. 4 and 5), while deletion of *LDP1* did not lead to any developmental effects. To our knowledge, this is the first study to describe LIM proteins as pathogenicity determinants in fungi.

Pax1 encodes a paxillin-like protein in *M. oryzae*. Previously, fungal paxillin-related proteins have been functionally identified in *S. cerevisiae*, where ScPxl1 localizes to sites of polarized growth and is required for selection and maintenance of polarized growth sites [25], [27]. In *S. pombe*, SpPxl1 is a component of the fission yeast actomyosin ring, which localizes to the division area and plays a role in formation and contraction of the actomyosin ring during cytokinesis [36], [28]. LIM domains are necessary for its function [28]. In *A. gossypii*, the paxillin-like protein AgPxl1 is necessary for apical branching and hyphal growth and localizes to emergence sites of new branches [29]. In addition, AgPxl1 also localizes to septa during cross wall formation but only temporarily in mature septa [29]. Recently, the conserved C-terminal LIM domains of AgPxl1 have been shown to be necessary for protein function and to contribute to tip localization [30]. We found that *M. oryzae* Pax1 is also crucial for hyphal growth (Fig. 2), which is similar to observation in the above fungal species. Moreover, the three LIM domains of Pax1 were essential for both its function and localization, but only when they were all present in the Pax1 protein (Fig. 8B).

Previously, co-immunoprecipitation (Co-IP) experiments have shown that SpPxl1 interacts directly with Rho1 in *S. pombe* and with Rho1-GDP (the inactive form of Rho1) in *S. cerevisiae* and *A. gossypii* [27], [28], [30]. However, we failed to observe any interactions between *M. oryzae* Pax1 and Rho GTPases in Y2H assays (Fig. S5). It is therefore not clear whether Pax1 can physically interact with the inactive form of Rho GTPases. It has been reported that the C-terminal LIM domains of animal paxillin were able to bind the tyrosine phosphatase PTP-PEST to target the protein to focal adhesions [46]. However, we did not find any interaction between *M*. *oryzae* Pax1 and PTP-PESTs in Y2H assays (Fig. S5). Therefore, to further understand the roles of Pax1 during fungal morphogenesis it will be necessary to carry out detailed in vivo interaction studies using co-immunoprecipitation to identify interacting partners.

*M*. *oryzae* Lrg1 belongs to a class of LIM proteins containing both RhoGAP and LIM domains. To date, only *S. cerevisiae* and *N. crassa* Lrg1 proteins have been functionally characterized in fungi. ScLrg1 shows a peak of expression during sporulation and plays a role during mating [31]. Disruption of ScLrg1 resulted in reduction of cell fusion, diploid formation and inhibition of mother-daughter separation [34], [35]. In *N. crassa*, NcLrg1 is essential for apical tip extension and for restricting excessive branch formation in sub-apical regions of hyphae and is also involved in determining the size of the hyphal compartments [37]. Like NcLrg1, we demonstrated that *M. oryzae* Lrg1 is also required for cell compartmentalization by regulating conidial shape and septation, and is necessary for normal growth, appressorium formation and pathogenicity.

Previously, domain functional analysis by site-directed mutagenesis has provided evidence that the three LIM domains and RhoGAP domain of *Neurospora* Lrg1 were both essential for its function of growth and septation, but only the LIM domains were crucial for localization of NcLrg1 protein to hyphal tips and septal pore [37]. Consistently, we found that Lrg1 expressed specifically at septal pores in hyphae and conidia, both Lrg1-LIM2 (the 2nd LIM) and Lrg1-RhoGAP affected function and localization of Lrg1 (Fig. 7 and 8). In addition, *S. cerevisiae* Lrg1 has been reported to specifically interact with the active form of Rho1 in Y2H analyses [32]. Another report also revealed that the RhoGAP domain of the ScLrg1 protein (containing its putative GAP domain and some flanking sequences) can interact with the mutant form of Rho1^Q68H/C206S^, a hyperactive derivative which mimics the GTP-bound form [33]. However, in this study, we did not detect any interactions between *M*. *oryzae* Lrg1 and Rho GTPases in Y2H assays (data not shown). Whether Lrg1 interacts with the active form of Rho1 and other Rho GTPases will be explored in the near future.

Rga1 also contains a RhoGAP domain, but deletion of *RGA1* and *LRG1* led to distinct phenotypic changes, although the two proteins displayed the same cellular localization to septal pores. In *S. cerevisiae*, ScRga1 interacts with Cdc42 and activates the pheromone-response pathway [36]. However, no interaction was detected between Rga1 and Cdc42 in *M*. *oryzae* (data not shown). Whether Rga1 is able to interact with the constitutively active Cdc42 GTPase remains unknown.

Determining the precise function of each LIM domain protein will require specific identification of their target proteins and interacting partners, which is currently underway.

## Materials and Methods

### Strains, culture conditions and molecular manipulations

Wild-type and recombinant strains of *M. oryzae* used in this study are listed in Table S1. Standard growth and storage procedures for fungal strains were performed, as described previously [47]. To prepare mycelial suspensions, mycelium was harvested from 48 h liquid CM cultures. *Escherichia coli* strain DH-5α was used for routine bacterial transformations and maintenance of all plasmids used in this study. Southern blot analysis was performed by the digoxigenin (DIG) high prime DNA labeling and detection starter Kit I (Roche, Mannheim, Germany). General procedures for nucleic acid analysis followed standard protocols [48].

### Construction of gene knockout vectors and generation of gene deletion mutants

Primers for constructing gene deletion vectors are listed in Table S2. Approximate 1 kb up- and down-stream region of each targeted gene and 1.5 kb *trpC-HPH* cassette were amplified from *M. oryzae* genome and cloned into pCB1003. Using a similar construction strategy, as described previously [49], targeted gene deletion vectors of the four LIM protein genes were constructed. *M. oryzae* protoplasts were prepared by digesting mycelium with Glucanex (Novozyme Switzerland AG) and harvested protoplasts diluted to 10^8^ cells/ml in STC buffer (0.6 M sorbitol, 10 mM Tris-HCl pH7.5, 10 mM CaCl_2_) for fungal transformation. Hygromycin resistant transformants were selected and gene deletion events analyzed by PCR amplification and confirmed by Southern blot.

### Gene complementation assays and functional analysis of various domains

To construct complementation vectors of *PAX1*, *LRG1* and *RGA1*, full length gene-coding sequence of each gene and promoter region (∼1.5 kb) was amplified and cloned into pGEM-T (Promega). A 1.4 kb GFP allele was amplified and ligated in-frame to the C-terminus of each gene. Finally, each resulting fragment containing promoter-ORF-GFP was cloned into pCB1532 [50] to generate the complementation vector. The resulting vectors were transformed into *Δpax1*, *Δlrg1* and *Δrga1* mutants, respectively. Transformants were screened for sulfonylurea resistance on BDCM.

Based on corresponding gene complementation vectors, LIM domain deletion vectors were constructed by overlapping extension PCR, as described previously [51]. Resulting vectors were transformed into *Δpax1* and *Δlrg1* mutants, respectively. Transformants were screened for sulfonylurea resistance on BDCM.

### Fungal growth, sporulation, appressorium development assays and genetic crosses

Vegetative growth was assessed by measurement of colony diameters in plate cultures of *M. oryzae* grown on CM medium at 25°C for 10 d. Osmotic, oxidative and cell wall integrity assays were carried out in CM agar supplemented with 1 M NaCl, 1.2 M sorbitol (Amresco), 50 µg/ml Congo Red (Sigma), 0.05% sodium dodecyl sulfate (SDS) and 5 mM H_2_O_2_ (Sigma), respectively. Inhibition rates (%) were calculated as follows: (D_0_−D_1_)/D_0_ ×100, D_0_ and D_1_ represent diameters of the cultures on CM medium at 25°C for 10 d with and without exogenous treatment, respectively. Conidiogenesis was analyzed by harvesting conidia from the surface of 15-day-old plate cultures and determining the concentration of resulting conidial suspension using a haemocytometer. Conidial shape and size were observed and measured by optical microscopy (Olympus, CX21). For appressorium formation and penetration assays, 20 µl conidial suspensions of 1×10^5^ spores/ml were dropped onto hydrophobic GelBond film and onion epidermis surfaces and cultured at 25°C for 24 h or 48 h. The percentage of conidia forming appressoria was determined by microscopic examination of at least 300 conidia or appressoria. Fertility assays were carried out by pairing Guy11 (*MAT1-2*), Ku70, Ku80 and isogenic mutants with the standard tester strain TH3 (*MAT1-1*) on oatmeal agar (OMA) plates, as described previously [52], [53]. Junctions between mated individuals were examined for the capacity to form perithecia. Each test was repeated at least three times.

### Pathogenicity assays

For barley infection assay, mycelium was prepared and used to inoculate 1-week-old cut barley leaves of cultivar Golden Promise. Mycelium was placed onto barley leaves and incubated in a humid chamber at 25°C. Disease lesions were examined at 7 d after inoculation. For rice infection assays, conidial suspensions were diluted in 0.2% gelatin to 1×10^5^ conidia/ml and 5 ml of each conidial suspension spray-inoculated onto 2-week-old rice seedlings of rice cultivar CO-39. For rice root infection assays, rice seeds were incubated at 28°C for 3 days to germinate and then transferred to plates contained 2% agar. Mycelium plugs were carefully placed on rice roots. Disease lesions were examined after 7 days of incubation. Each test was repeated three times.

### Quantitative RT-PCR analysis

Quantitative RT-PCR (qRT-PCR) was performed consistent with the guidelines for minimum information for publication of quantitative Real-Time PCR experiments (MIQE) [54]. Total RNA was isolated from mycelium using RNAiso Plus reagent (TaKaRa) and used to synthesize first-strand cDNA using PrimeScript®RT (TaKaRa). RT-PCR was performed with SYBR® *Premix Ex Taq*™ Kit (TaKaRa) using the BIO-RAD CFX96™ Real-Time System. Primers used for qRT-PCR assays are listed in Table S2. The relative expression level of each gene was calculated as the 2^−ΔΔCT^ method [55] with the histone gene MGG_01160.6 as reference. Mean and standard deviation were determined with data from three replicates.

### Yeast two-hybrid (Y2H) assay

Y2H assay was performed according to the BD Matchmaker Library Construction & Screening Kits instructions (Clontech, PaloAlto, CA, USA). Full-length cDNAs of each candidate gene were amplified with primers listed in Table S2. The Pax1 cDNA was cloned into bait plasmid pGBK and cDNAs of Rho1-5, Cdc42, Rac1 and PTP-PEST1-3 were respectively cloned into prey plasmid pGAD. The resulting bait vector and each prey vector were co-transformed into yeast strain AH109. Growth of yeast transformants was determined on SD-Trp-Leu-His-Ade medium.

## Supporting Information

Figure S1 **Gene deletion of LIM protein genes and confirmation.** **A.** *LDP1* deletion strategy (left) and confirmation by Southern blot (right). Genomic DNA was digested with *Xba*I and probed with upstream flanking sequence of *LDP1*. Lane 1, wild type strain; Lane 2 and 3, *Δldp1*; lane 4 and 5, ectopic transformants. X, *Xba*I; P, *Pst*I; Spe, *Spe*I; E, *Eco*RI; N, *Not*I. **B.** *PAX1* deletion strategy (left) and confirmation by Southern blot (right). Genomic DNA was digested with *Hin*dIII and probed with upstream flanking sequence of *PAX1*. Lane 1, wild type strain; Lane 2 to 4, *Δpax1*; lane 5, ectopic transformant. H, *Hin*dIII; P, *Pst*I; Spe, *Spe*I; E, *Eco*RI. **C.** *RGA1* deletion strategy (left) and confirmation by Southern blot (right). Genomic DNA was digested with *Sac*I and probed with upstream flanking sequence of *RGA1*. Lane 1, wild type strain; Lane 2 to 4, *Δrga1*; lane 5, ectopic transformant. Sac, *Sac*I; P, *Pst*I; Spe, *Spe*I; E, *Eco*RI. **D.** *LRG1* deletion strategy (left) and confirmation by Southern blot (right). Genomic DNA was double-digested with *Sal*I and *Kpn*I and probed with downstream flanking sequence of *LRG1*. Lane 1, wild type strain; Lane 2 and 3, *Δlrg1*; lane 4, ectopic transformant. K, *Kpn*I; Sal, *Sal*I; P, *Pst*I; Spe, *Spe*I; E, *Eco*RI. Asterisk represents restriction sites introduced or derived from vectors.
(TIF)

Figure S2 **Fertility assay of LIM protein mutants.** The four LIM protein mutants, *Δldp1* (LD17), *Δpax1* (LP55), *Δlrg1* (LR80) and *Δrga1* (LG25) were crossed with TH3 strain, respectively. Numerous black perithecia were observed at the junction of different crosses, indicating that these LIM proteins are not required for sexual reproduction by *M. oryzae*.
(TIF)

Figure S3 **Patterns of** ***LRG1*** **expression during appressorium development.** Conidia of the strain RC38 (*Δlrg1:LRG1*) was allowed to germinate on hydrophobic GelBond film surfaces. Photographs were taken at various time intervals. BF  =  bright field. Scale bar  =  10 µm.
(TIF)

Figure S4 **Patterns of** ***PAX1*** **expression during appressorium development.** Conidia of the strain PC20 (*Δpax1:PAX1*) was allowed to germinate on hydrophobic GelBond film surfaces. Photographs were taken at various time intervals. BF  =  bright field. Scale bar  =  10 µm.
(TIF)

Figure S5 **Y2H assay to detect interactions between Pax1 and its partners.** No direct interaction was detected between Pax1 and partners. Rho1, MGG_07176; Rho2, MGG_02457; Rho3, MGG_10323; Rho4, MGG_03901; Rho5, MGG_03295; Cdc42, MGG_00466; Rac1, MGG_02731; PTP-PEST1, MGG_01376; PTP-PEST2, MGG_00912; PTP-PEST3, MGG_07602.
(TIF)

Table S1 **Wild-type and recombinant strains of** ***M. oryzae*** **used in this study.**
(DOC)

Table S2 **PCR primers used in this study.**
(DOC)

Table S3 **Inhibitory effects of various chemicals on vegetative growth of** ***Δpax1*** **and** ***Δlrg1*** **mutants.**
(DOC)
