# Supplementary figures and images for: Characterisation of Four LIM Protein-Encoding Genes Involved in Infection-Related Development and Pathogenicity by the Rice Blast Fungus Magnaporthe oryzae
Source: PLoS One. 2014 Feb 5;9(2):e88246. doi: 10.1371/journal.pone.0088246 (PMC3914944; doi:10.1371/journal.pone.0088246)

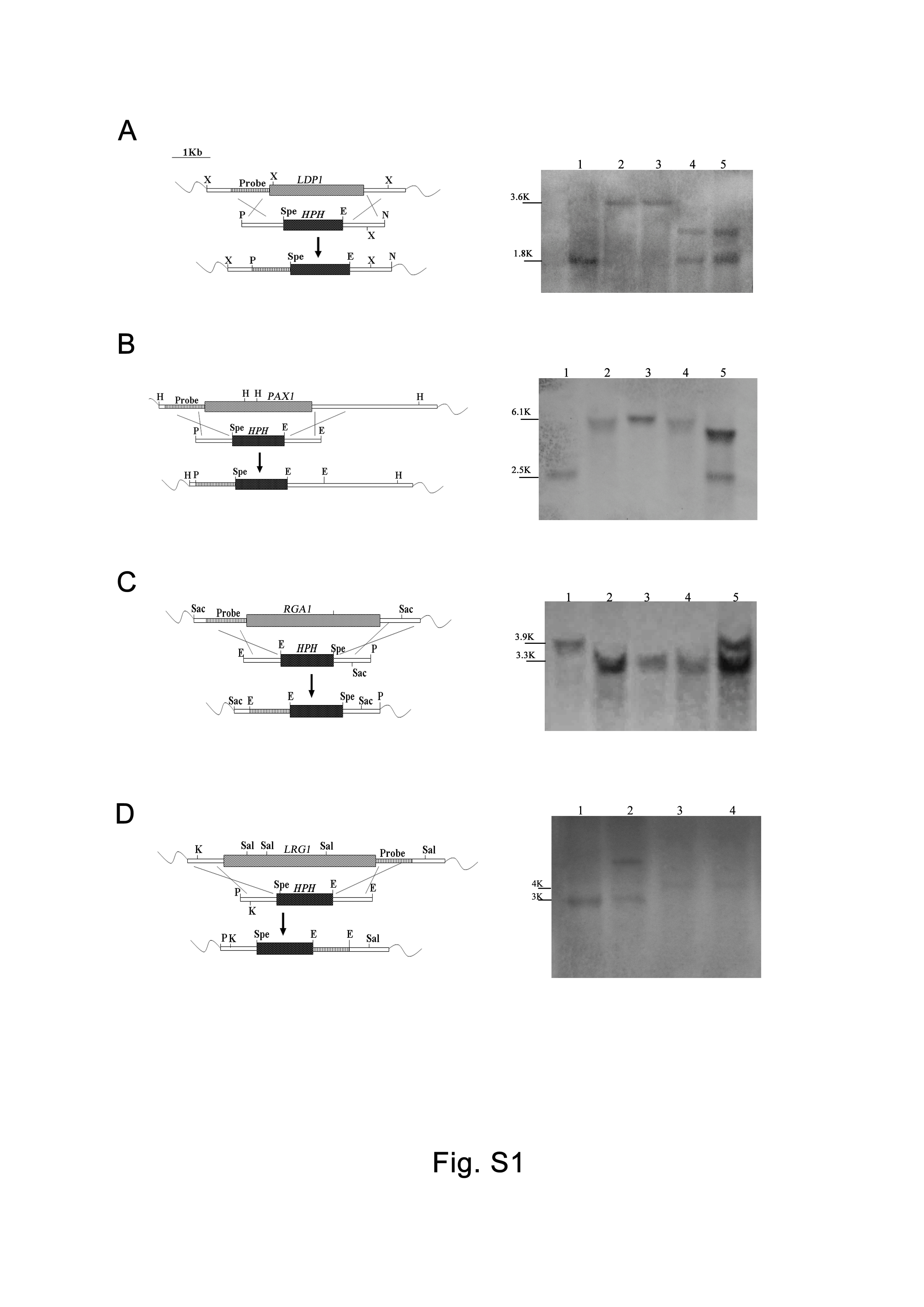

Supplement: Figure S1 — Gene deletion of LIM protein genes and confirmation. A. LDP1 deletion strategy (left) and confirmation by Southern blot (right). Genomic DNA was digested with XbaI and probed with upstream flanking sequence of LDP1. Lane 1, wild type strain; Lane 2 and 3, Δldp1; lane 4 and 5, ectopic transformants. X, XbaI; P, PstI; Spe, SpeI; E, EcoRI; N, NotI. B. PAX1 deletion strategy (left) and confirmation by Southern blot (right). Genomic DNA was digested with HindIII and probed with upstream flanking sequence of PAX1. Lane 1, wild type strain; Lane 2 to 4, Δpax1; lane 5, ectopic transformant. H, HindIII; P, PstI; Spe, SpeI; E, EcoRI. C. RGA1 deletion strategy (left) and confirmation by Southern blot (right). Genomic DNA was digested with SacI and probed with upstream flanking sequence of RGA1. Lane 1, wild type strain; Lane 2 to 4, Δrga1; lane 5, ectopic transformant. Sac, SacI; P, PstI; Spe, SpeI; E, EcoRI. D. LRG1 deletion strategy (left) and confirmation by Southern blot (right). Genomic DNA was double-digested with SalI and KpnI and probed with downstream flanking sequence of LRG1. Lane 1, wild type strain; Lane 2 and 3, Δlrg1; lane 4, ectopic transformant. K, KpnI; Sal, SalI; P, PstI; Spe, SpeI; E, EcoRI. Asterisk represents restriction sites introduced or derived from vectors. (TIF) [file pone.0088246.s001.tif]

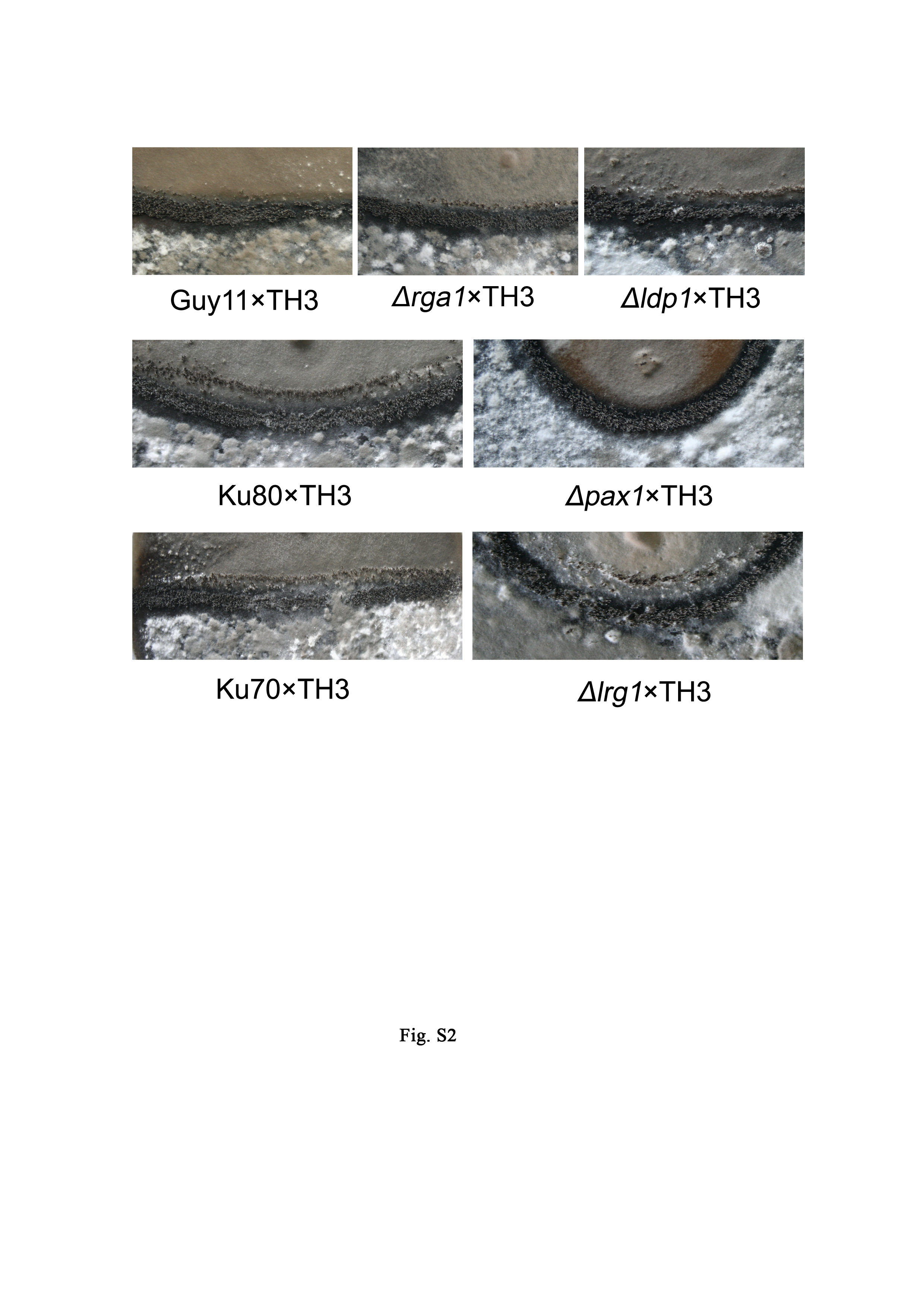

Supplement: Figure S2 — Fertility assay of LIM protein mutants. The four LIM protein mutants, Δldp1 (LD17), Δpax1 (LP55), Δlrg1 (LR80) and Δrga1 (LG25) were crossed with TH3 strain, respectively. Numerous black perithecia were observed at the junction of different crosses, indicating that these LIM proteins are not required for sexual reproduction by M. oryzae. (TIF) [file pone.0088246.s002.tif]

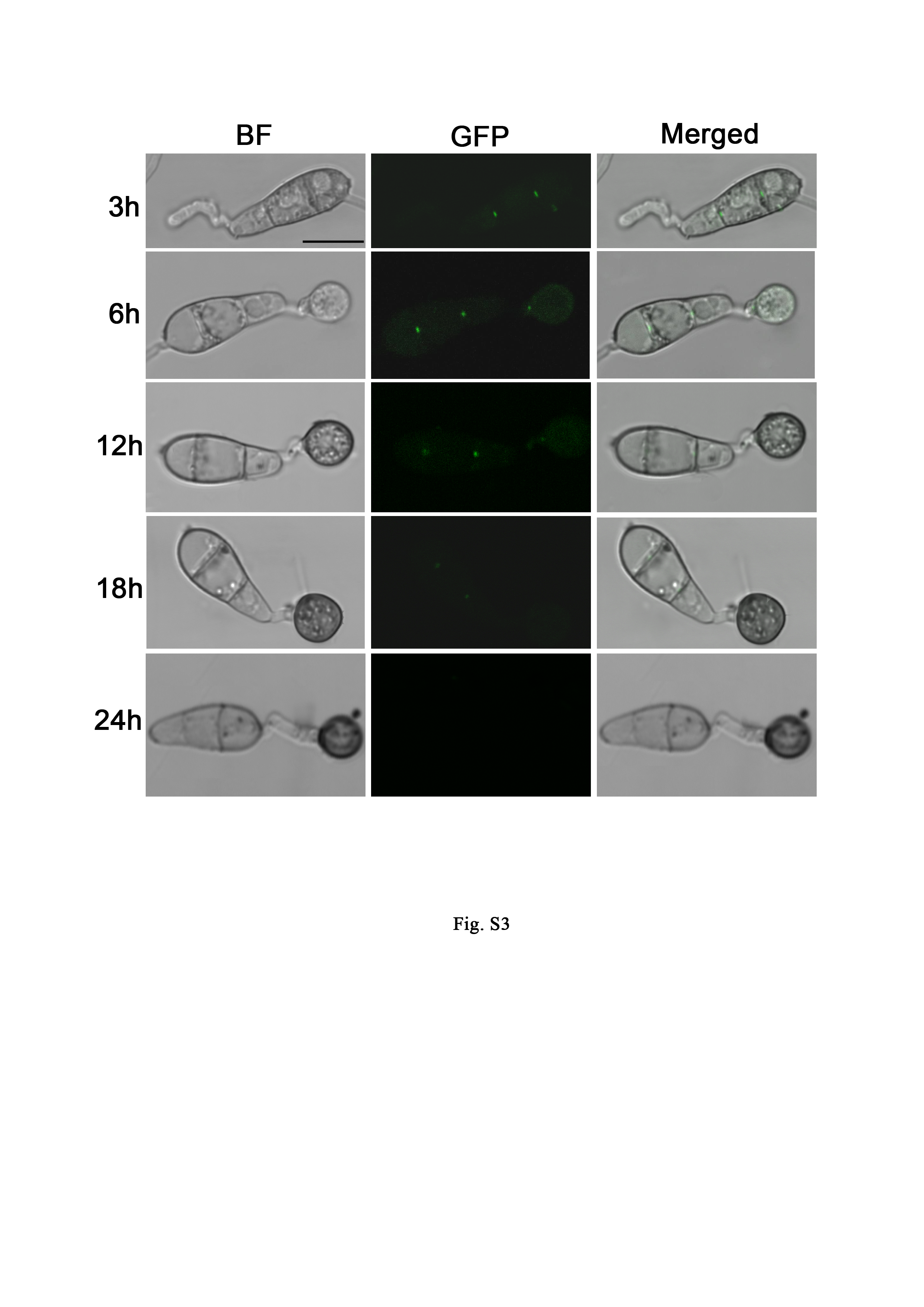

Supplement: Figure S3 — Patterns of LRG1 expression during appressorium development. Conidia of the strain RC38 (Δlrg1:LRG1) was allowed to germinate on hydrophobic GelBond film surfaces. Photographs were taken at various time intervals. BF = bright field. Scale bar = 10 µm. (TIF) [file pone.0088246.s003.tif]

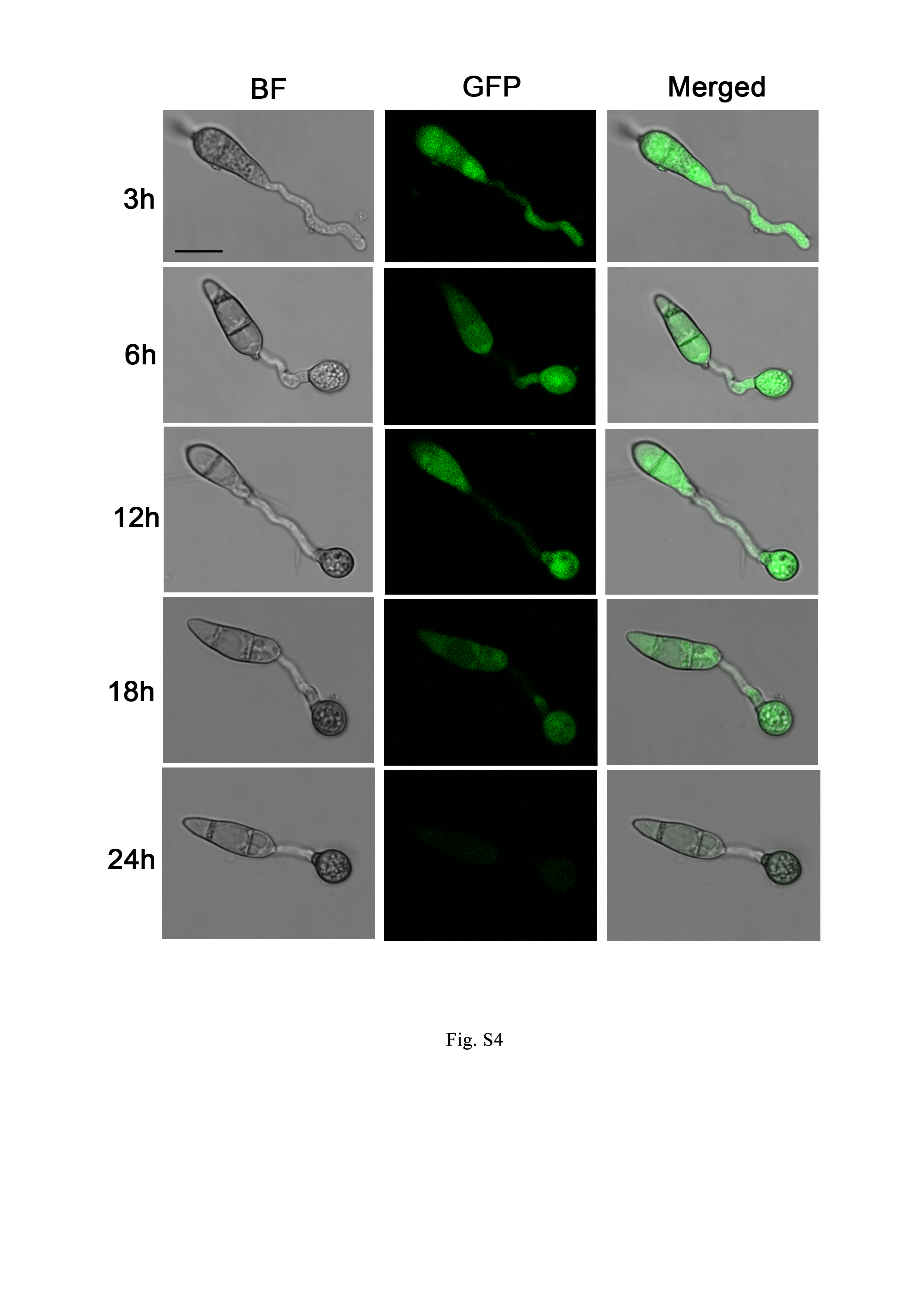

Supplement: Figure S4 — Patterns of PAX1 expression during appressorium development. Conidia of the strain PC20 (Δpax1:PAX1) was allowed to germinate on hydrophobic GelBond film surfaces. Photographs were taken at various time intervals. BF = bright field. Scale bar = 10 µm. (TIF) [file pone.0088246.s004.tif]

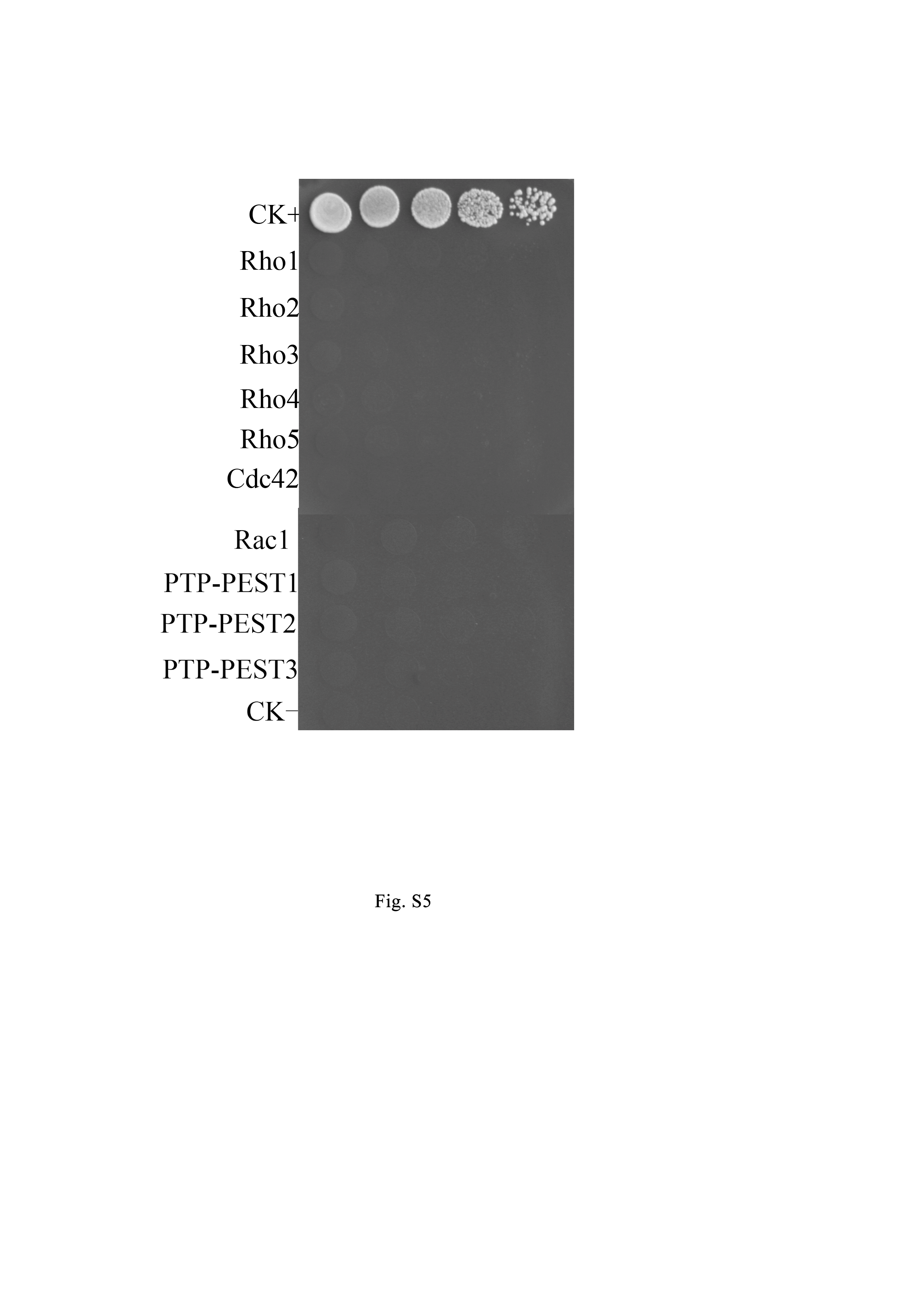

Supplement: Figure S5 — Y2H assay to detect interactions between Pax1 and its partners. No direct interaction was detected between Pax1 and partners. Rho1, MGG_07176; Rho2, MGG_02457; Rho3, MGG_10323; Rho4, MGG_03901; Rho5, MGG_03295; Cdc42, MGG_00466; Rac1, MGG_02731; PTP-PEST1, MGG_01376; PTP-PEST2, MGG_00912; PTP-PEST3, MGG_07602. (TIF) [file pone.0088246.s005.tif]
