# Supplementary material for: Characterisation of Four LIM Protein-Encoding Genes Involved in Infection-Related Development and Pathogenicity by the Rice Blast Fungus Magnaporthe oryzae
Source: PLoS One. 2014 Feb 5;9(2):e88246. doi: 10.1371/journal.pone.0088246 (PMC3914944; doi:10.1371/journal.pone.0088246)
Supplement: Table S1 — Wild-type and recombinant strains of M. oryzae used in this study. (DOC) [file pone.0088246.s006.doc]

**Table S1. Wild-type and recombinant strains of *M. oryzae* used in this study**

| ***Strain*** | ***Mutant*** | ***Brief description*** | Reference |
| --- | --- | --- | --- |
| Guy11 | / | Wild type (*MAT1-2*) | Leung *et al*., 1988 |
| TH3 | / | Wild-type (*MAT1-1*) | Dixon *et al*., 1999 |
| Ku80 | *Δku80* | Deletion of *KU80* in Guy11(*MAT1-2*) | Villalba *et al*., 2008 |
| Ku70 | *Δku70* | Deletion of *KU70* in Guy11(*MAT1-2*) | Villalba *et al*., 2008 |
| LP55, LP62, LP68 | *Δpax1* | *Δpax1* mutants of Ku80 | This study |
| PC20 | *Δpax1:PAX1* | LP55 (*Δpax1*) transformed with *PAX1*-*GFP* | This study |
| PCT15 | *PAX1^ΔLIM1^* | LP55 transformed with *PAX1^ΔLIM1^*-*GFP* | This study |
| PCT18 | *MoPAX1^ΔLIM2^* | LP55 transformed with *MoPAX1^ΔLIM2^*-*GFP* | This study |
| PCT32 | *PAX1^ΔLIM3^* | LP55 transformed with *PAX1^ΔLIM3^*-*GFP* | This study |
| PCT20 | *PAX1^ΔLIM1+2+3^* | LP55 transformed with *PAX1^ΔLIM1+2+3^*-*GFP* | This study |
| LR80, LR95, LR98 | *Δlrg1* | *Δlrg1* mutants of Ku70 | This study |
| RC38 | *Δlrg1:LRG1* | LR80 (*Δlrg1:LRG1*) transformed with *LRG1*-*GFP* | This study |
| RCT35 | *LRG1^ΔLIM1^* | LR80 transformed with *LRG1^ΔLIM1^*-*GFP* | This study |
| RCT26 | *LRG1^ΔLIM2^* | LP55 transformed with *LRG1^ΔLIM2^*-*GFP* | This study |
| RCT32 | *LRG1^ΔLIM3^* | LP55 transformed with *LRG1^ΔLIM3^*-*GFP* | This study |
| RCT28 | *LRG1^ΔLIM1+2+3^* | LP55 transformed with *LRG1^ΔLIM1+2+3^*-*GFP* | This study |
| LG25, LG30, LG50 | *Δrga1* | *Δrga1* mutants of Guy11 | This study |
| GC22 | *Δrga1:RGA1* | LG25 (*Δrga1*) transformed with *RGA1*-*GFP* | This study |
| LD17, LD22, LD29 | *Δldp1* | *Δldp1* deletion mutants of Guy11 | This study |
| **AD27, AD33** | *Δldp1Δrga1* | *Δldp1Δrga1* mutants | This study |
