# Supplementary material for: Characterisation of Four LIM Protein-Encoding Genes Involved in Infection-Related Development and Pathogenicity by the Rice Blast Fungus Magnaporthe oryzae
Source: PLoS One. 2014 Feb 5;9(2):e88246. doi: 10.1371/journal.pone.0088246 (PMC3914944; doi:10.1371/journal.pone.0088246)
Supplement: Table S2 — PCR primers used in this study. (DOC) [file pone.0088246.s007.doc]

**Table S2** Primers used in this study

| **Name** | **Sequence（5’→3’）** | **Introduced restriction site** | **Brief description** |
| --- | --- | --- | --- |
| LDP1-up-Pst1-F | CTGCAGCATGGGCATTTAACGGAG | *Pst*I | *LDP1* deletion |
| LDP1-up-Spe1-R | ACTAGTTTGTGTCTCCCGGTTAGTCC | *Spe*I |
| LDP1-down-EcoR1-F | GAATTCATGCCCTCCAAACCTCTA | *Eco*RI |
| LDP1-down-Not1-R | GCGGCCGCTCAAATCGGTCAACTAACGC | *Not*I |
| PAX1-up-Pst1-F | CTGCAGCAAGGTAAGGTAAGGTAAGG | *Pst*I | *PAX1* deletion |
| PAX1-up-Spe1-R | ACTAGTGGTTTGCAGCTATTCGGAGGATT | *Spe*I |
| PAX1-down-EcoR1-F | GAATTCAGTTTGAGAGGGTTGGGTTCACG | *Eco*RI |
| PAX1-down-EcoR1-R | GAATTCAAGGGTCACCAGGTGGAGGC | *Eco*RI |
| RGA1-up-EcoR1-F | GAATTCCATGACGACCGCCCTTGAAC | *Eco*RI | *RGA1* deletion |
| RGA1-up-EcoR1-R | GAATTCGGTGGTTATGTTGCGCTGGG | *Eco*RI |
| RGA1-down-Spe1-F | ACTAGTGATAACCTCTGATCCCGATGG | *Spe*I |
| RGA1-down-Pst1-R | CTGCAGGCCGGCCTACTTCCAA | *Pst*I |
| LRG1-up-Pst1-F | CTGCAGGAATAGTTTAATGTATCGTA | *Pst*I | *LRG1* deletion |
| LRG1-up-Spe1-R | ACTAGTGCCGCTTTTTGATGTGGCG | *Spe*I |
| LRG1-down-EcoR1-F | GAATTCTCCCTCTACTTTGGTTGGCT | *Eco*RI |
| LRG1-down-EcoR1-R | GAATTCCTTATTTCCCATCCCAACTC | *Eco*RI |
| HPH-F | TATTGAAGGAGCATTTTTGG | - | *HPH* gene |
| HPH-R | GCTCTTGTTCGGTCGGCATC | - |
| LRG1-Pro-Not1-F | GCGGCCGCTCTACTAGTAATGAAGGTGTGG | *Not*I | *LRG1* complementation |
| LRG1-Orf-Kpn1-R | GGTACCGCCAGTTCCAGTAACACCAACAGAG | *Kpn*I |
| PAX1-Pro-Xba1-F | TCTAGAGGTTGGTATGTAGGTGCGCAGCTTGG | *Xba*I | *PAX1* complementation |
| PAX1-Orf-Sal1-R | GTCGACGCCCTTCAGCTCCCTCGCTCGACA | *Sal*I |
| RGA1-Pro-Not1-F | GCGGCCGCGACGTTTAATCTAATCCTTTGC | *Not*I | *RGA1* complementation |
| RGA1-Orf-EcoR1-R | GAATTCAGCCTCTTCGAAGATGATGTGAC | *Eco*RI |
| GFP-Kpn1-F | GGTACCACTTAACGTTACTGAAATC | *Kpn*I | GFP gene |
| GFP-Kpn1-R | GGTACCGGATTACCTCTAAACAAGTG | *Kpn*I |
| LRG1-Lim1out-F | GGTAGCAGAGGAGGCCGATGCGCAGCTGCCCG | - | *LRG1* LIM1 deletion |
| LRG1-Lim1out-R | CGGGCAGCTGCGCATCGGCCTCCTCTGCTACC | - |
| LRG1-Lim2out-F | CGTTGCACTTTTGTGCGAGGAGGCCTAGACGA | - | *LRG1* LIM2 deletion |
| LRG1-Lim2out-R | TCGTCTAGGCCTCCTCGCACAAAAGTGCAACG | - |
| LRG1-Lim3out-F | ATGGGGGCGCATTGTCCTGATCTGAGTCGTTA | - | *LRG1* LIM3 deletion |
| LRG1-Lim3out-R | TAACGACTCAGATCAGGACAATGCGCCCCCAT | - |
| LRG1-RhoGAPout-F | CCAGAGTAAGGGCCTTGATGCGTAGCGTTCCG | - | *LRG1* RhoGAP deletion |
| LRG1-RhoGAPout-R | CGGAACGCTACGCATCAAGGCCCTTACTCTGG | - |
| PAX1-Lim1out-R | GACTGATCTCAAGGGCGCGAGCCGTAGCCCGG | - | *PAX1* LIM1 deletion |
| PAX1-Lim1out-F | CCGGGCTACGGCTCGCGCCCTTGAGATCAGTC | - |
| PAX1-Lim2out-R | TCTCCGTTCGCTTGGTCTTGGGCGCAAACTGC | - | *PAX1* LIM2 deletion |
| PAX1-Lim2out-F | GCAGTTTGCGCCCAAGACCAAGCGAACGGAGA | - |
| PAX1-Lim3out-R | TCAGCTCCCTCGCTCGCTTGGGCGCCCTCCTC | - | *PAX1* LIM3 deletion |
| PAX1-Lim3out-F | GAGGAGGGCGCCCAAGCGAGCGAGGGAGCTGA | - |
| PAX1-N-termini-R | GCGAGCCGTAGCCCGG | - | *PAX1* LIM1+2+3 deletion |

**Continued**

| Histone-MG01160-F | CGCAAGATTCTTCGTGACAA | - | Reference gene for qRT-PCR |
| --- | --- | --- | --- |
| Histone-MG01160-R | TCTTGGCGTGCTCTGTGTAG | - |
| CHS1-MG01802-F | AGGGAGAGACCGATGTTCCT | - | *CHS1* qRTPCR |
| CHS1-MG01802-R | AGCTGGACGTGGAAGAAGAA | - |
| CHS2-MG04145-F | ACCACTTGTCCCTCAACCAG | - | *CHS2* qRT-PCR |
| CHS2-MG04145-R | CACGATCCTCCAAGAGCTTC | - |
| CHS3-MG09551-F | CGACCAGCTTCAACTTCACA | - | *CHS3* qRT-PCR |
| CHS3-MG09551-R | GGAGTCTGAGCTTCGTTTGG | - |
| CHS4-MG09962-F | GATTGCAAACAAGCGAGACA | - | *CHS4* qRT-PCR |
| CHS4-MG09962-R | TGTCGACGACGTTTTCAGAG | - |
| CHS5-MG13014-F | CTAGTGTGGGCCATCACCTT | - | *CHS5* qRT-PCR |
| CHS5-MG13014-R | GCTTAACCTCATCGCGAGTC | - |
| CHS6-MG13013-F | TATGCGCTACGATGACAAGC | - | *CHS6* qRT-PCR |
| CHS6-MG13013-R | CGAGTAAACCTTGCCCATGT | - |
| CHS7-MG06064-F | AAACTCGAGGGACATGTTGG | - | *CHS7* qRT-PCR |
| CHS7-MG06064-R | CCTCCTGAACGCAGAGAAAC | - |
| GLS1-MG00856-F | GGCGTATGTACCTCGTCGTT | - | *GLS1* qRT-PCR |
| GLS1-MG00856-R | AAGAGGAAAGTCGCCACAGA | - |
| Rho1RT-EcoR1-F | GAATTCATGGCCGAAATCCGCCGCAAG | *Eco*RI | Rho1 for Y2H |
| Rho1RT-BamH1-R | GGATCCTTAGAGGATGAGGCACTTCT | *BamH*I |
| Rho2RT-EcoR1-F | GAATTCATGGCGGCCGCAAATAATCAG | *Eco*RI | Rho2 for Y2H |
| Rho2RT-BamH1-R | GGATCCCTATAGGATCACACAGCATC | *BamH*I |
| Rho3RT-BamH1-F | GGATCCATGCCTTTATCGCTTTGC | *BamH*I | Rho3 for Y2H |
| Rho3RT-Xho1-R | CTCGAGCTACATGACGGTGCACTTGC | *Xho*I |
| Rho4RT-EcoR1-F | GAATTCATGGCTTCGCAATACCAGTAT | *Eco*RI | Rho4 for Y2H |
| Rho4RT-BamH1-R | GGATCCTCACAAGATATTGCACCTCG | *BamH*I |
| Rho5RT-EcoR1-F | GAATTCATGATGGACCTAGACTGGC | *Eco*RI | Rho5 for Y2H |
| Rho5-BamH1-R | GGATCCCTATGCTGTGCTAGGCCTGT | *BamH*I |
| Cdc42RT-EcoR1-F | GAATTCATGGTGGTTGCAACGATT | *Eco*RI | Cdc42 for Y2H |
| Cdc42RT-BamH1-R | GGATCCTCAAAGGATCAGGCACTT | *BamH*I |
| Rac1RT-EcoR1-F | GAATTCATGGCCGCCCCTGGGGTTCA | *Eco*RI | Rac1 for Y2H |
| Rac1RT-BamH1-R | GGATCCTCACAGAATGGTGCACTTTG | *BamH*I |
| PTP-PEST1-RT-F | GAATTCGGCATTGAGAAAGGGGACAAG | *Eco*RI | PTP-PEST1 for Y2H |
| PTP-PEST1-RT-F | GGATCCCTCAAGCACAGTCTCGTAGC | *BamH*I |
| PTP-PEST2-RT-F | GAATTCACCAGCACGACAGTTAAGAAGC | *Eco*RI | PTP-PEST2 for Y2H |
| PTP-PEST2-RT-F | GGATCCCTTGCGCATGACCTGGTAGATA | *BamH*I |
| PTP-PEST3-RT-F | GAATTCATGGCAAAAAGCAAGCCGGC | *Eco*RI | PTP-PEST3 for Y2H |
| PTP-PEST3-RT-R | GGATCCTCATTGTTGCTTGCCCTGTG | *BamH*I |
| Pax1RT-EcoRI-F | GAATTCATGTTTGCTCGAGGGAAGTC | *Eco*RI | Pax1 for Y2H |
| Pax1RT-BamHI-R | GGATCCTCAGCCCTTCAGCTCCCT | *BamH*I |

The introduced restriction site was underlined.
