# Supplementary material for: Characterisation of Four LIM Protein-Encoding Genes Involved in Infection-Related Development and Pathogenicity by the Rice Blast Fungus Magnaporthe oryzae
Source: PLoS One. 2014 Feb 5;9(2):e88246. doi: 10.1371/journal.pone.0088246 (PMC3914944; doi:10.1371/journal.pone.0088246)
Supplement: Table S3 — Inhibitory effects of various chemicals on vegetative growth of Δpax1 and Δlrg1 mutants. (DOC) [file pone.0088246.s008.doc]

**Table S3. Inhibitory effects of various chemicals on vegetative growth of *Δpax1* and *Δlrg1* mutants**

| **Strain** | **NaCl**  **(1 M)** | **Sorbitol**  **(1.2 M)** | **CR**  **(100 μg/ml)** | **SDS**  **(0.05%)** | **H_2_O_2_**  **(5 mM)** |
| --- | --- | --- | --- | --- | --- |
| Ku80 | 60.0±1.5a | 28.4±0.4a | 27.8±1.9a | 61.51±2.26a | 9.7±0.3a |
| *Δpax1* | 39.3±1.4b | 27.4±1.3a | 1.2±0.5b | 45.0±2.50c | 9.5±1.8a |
| Ku70 | 58.3±2.3a | 29.0±2.8a | 26.9±5.3a | 64.3±4.6a | 8.9±1.9a |
| *Δlrg1* | 19.6±1.8c | 26.4±2.2a | 10.7±4.4b | 52.33±1.91b | 6.8±1.5a |

Inhibition rate (%) was calculated from three independent experiments as described in Materials and Methods. CR, Congo Red; SDS, sodium dodecyl sulfate. Different letters indicated significant difference at P-value of 0.05.
